# Global transcriptome analysis of subterranean pod and seed in peanut (*Arachis hypogaea* L.) unravels the complexity of fruit development under dark condition

**DOI:** 10.1038/s41598-020-69943-7

**Published:** 2020-08-03

**Authors:** Hao Liu, Xuanqiang Liang, Qing Lu, Haifen Li, Haiyan Liu, Shaoxiong Li, Rajeev Varshney, Yanbin Hong, Xiaoping Chen

**Affiliations:** 1 Guangdong Provincial Key Laboratory of Crop Genetic Improvement, Crops Research Institute, Guangdong Academy of Agricultural Sciences (GDAAS), Guangzhou, 510640 China; 2 International Crops Research Institute for the Semi-Arid Tropics (ICRISAT), Patancheru, 502324 India

**Keywords:** Plant sciences, Plant physiology

## Abstract

Peanut pods develop underground, which is the most salient characteristic in peanut. However, its developmental transcriptome remains largely unknown. In the present study, we sequenced over one billion transcripts to explore the developmental transcriptome of peanut pod using Illumina sequencing. Moreover, we identified and quantified the abundances of 165,689 transcripts in seed and shell tissues along with a pod developmental gradient. The dynamic changes of differentially expressed transcripts (DETs) were described in seed and shell. Additionally, we found that photosynthetic genes were not only pronouncedly enriched in aerial pod, but also played roles in developing pod under dark condition. Genes functioning in photomorphogenesis showed distinct expression profiles along subterranean pod development. Clustering analysis unraveled a dynamic transcriptome, in which transcripts for DNA synthesis and cell division during pod expansion were transitioning to transcripts for cell expansion and storage activity during seed filling. Collectively, our study formed a transcriptional baseline for peanut fruit development under dark condition.

## Introduction

Peanut (*Arachis hypogaea* L.) is a globally cultivated crop for edible oil and protein production^1^, the fruit of which shows a distinctive development pattern compared with other legume species, “Aerial Flower, Subterranean Fruit”. Following fertilization, peanut gynophore elongates to form a special geotropic organ (called peg), and the peg harboring embryo continues to grow and push the developing pod into the soil for underground pod. Once penetration into soil, pod formation and embryonic differentiation occur to induce the seed production^2^. The subterranean fructification is the most prominent characteristic of seed production, and therefore it has the biologically important value for studying organogenesis and evolution^3^. Importantly, studies on peanut pod-seed development are of significance for exploring mechanism underlying the plant reproductive development and crop improvement under dark condition.

Recently, transcriptome technology opens new opportunities for mapping and quantifying the expressions of genes related to important crop agronomic traits. Transcriptomics have matured to the point where complex gene regulatory networks consisting of mRNA expression, transcription factors (TFs), small RNA and downstream target genes aid in elucidating complex developmental processes^4^. Understanding the global expression profiling during peanut pod-seed development, and redefining their transcriptome will provide crucial information for illustrating the fruit development under dark condition. In peanut, RNA-seq has been utilized to unravel transcriptome changes over early pod development and to identify candidate genes for oil accumulation pathways in the past years^5–7^.

The progress achieved in peanut transcriptome analysis has improved the understanding of expression patterns and their relation to function and regulation over development and under various stresses. Several studies on pod development have focused on only particular developmental stage^5–7^. However, these studies do not investigate the whole developmental stage of pod-seed simultaneously, and can not reflect the detailed links of transcriptomic commonalities and differences between the seed and shell (pod wall). Although some mechanisms of pod-seed development are common to all cells, major differences exist in strategies adopted by peanut to cope with its own developmental characteristics^8^. Presently, it remains difficult to find answers for questions at the molecular level, such as why and how peanut bears fruit underground. Peanut fruit undergoes aerial and subterranean development, which can be further partitioned into aerial elongation, subterranean pod expansion and seed filling, followed by seed desiccation^9^. For these complex developmental events, it is necessary to perform a comprehensive study covering the entire pod development.

In the present study, we generated an extensive transcriptional map for the entire pod-seed developmental stage in peanut by RNA-seq. We sequenced 20 separated seed and shell samples representing 11 distinct stages of pod development in order to extend our knowledge on pod expansion, seed filling and desiccation under dark conditions. Taken together, our data could serve as a valuable resource for transcriptome studies related to peanut pods.

## Results

### Phenotype characteristics of peanut pod (shell and seed) development

Peanut pod (shell and seed) development contained the typical feature of “Aerial Flower, Subterranean Fruit”, which firstly formed the gravity-guided aerial peg (P0) by self-pollination. Vertically growing subterranean peg further (P1) rapidly expanded self-volume to transform into pod under the subterranean dark condition, but from the aerial into the underground, peg diameter was not changed basically (P0–P1). Peanut shell seemed like a baby room, which was quickly constructed during the stage of P2–P6 (pod expansion), and the shell size was increased approximately six-folds by measuring the diameter values (P2: 2.0–4.0 mm, P6: 13.0–16.5 mm). However, peanut shell gradually stopped to expand self-volume from the stage of P6 until the terminal mature, and this behavior aimed to prepare an advantageous condition for seed development (Table S1). Subsequently, peanut seed initially grew rapidly at the time-point of P6 stage that represented a demarcation point of shell stop expansion, and the seed size was not increased until P10 (seed mature) stage (Table S1). Meanwhile, the entire period of peanut embryo development could also be clearly divided into two distinctive phases, and P6 was still the demarcation point by observing the light micrographs of developing embryo. At the stage of P0–P6, embryo grown with cotyledons appeared and elongated (Fig. S1A), and first and second pairs of leaf primordia mainly appeared at the phase of seed filled with bigger size (P7–P10) (Fig. S1B), while the cell size of pod shell was mainly increased quickly at the early stage (Fig. S1C). Totally, the development characteristics of peanut pod were different from other legume species, which probably adopted an optimal energy saving protocol to promote the fruit development.

### Transcriptome landscape of peanut pod development

**Figure 1:**
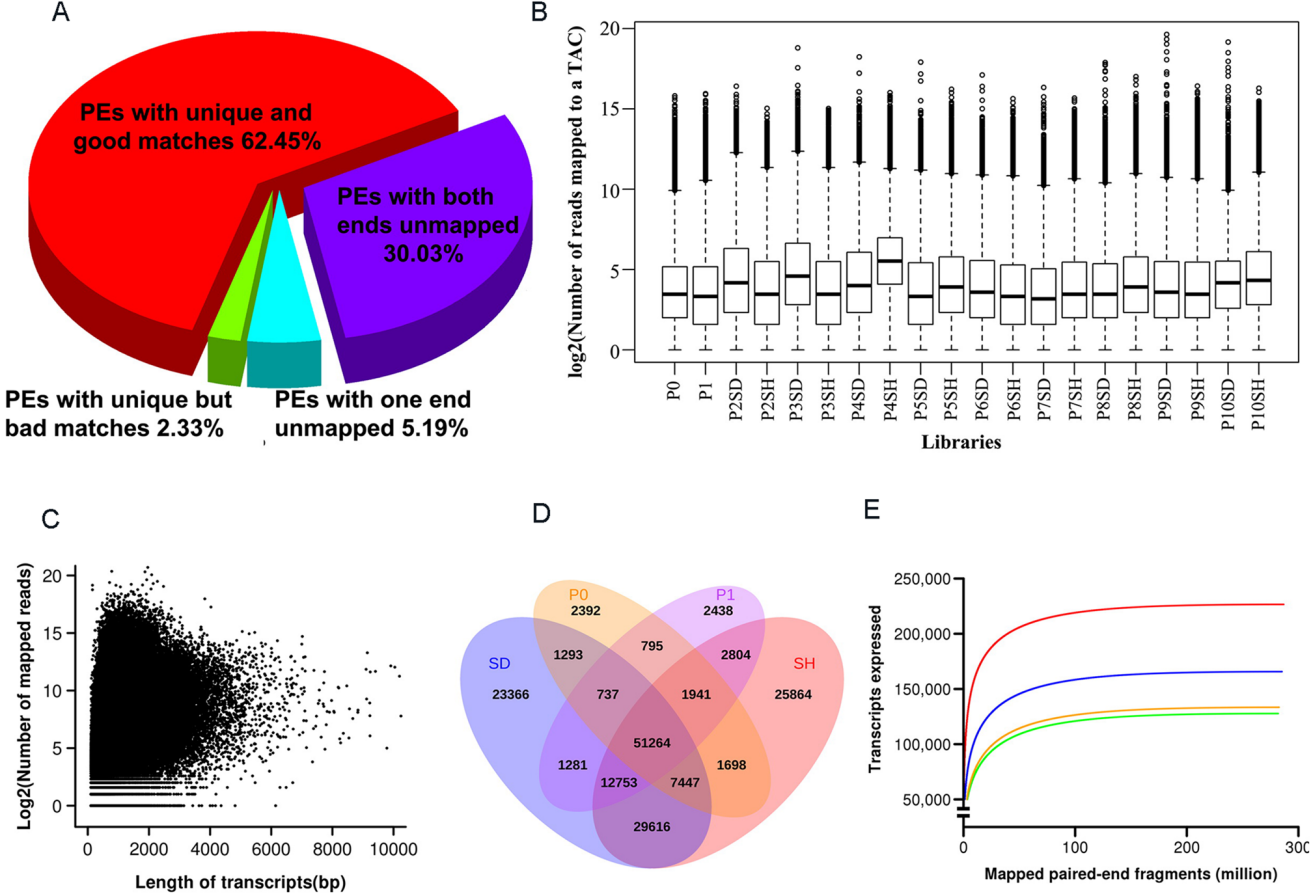
RNA-seq analysis of peanut pod transcriptome. (**A**) Overall mapping results of PEs for all libraries referring to the reference transcriptome (AHGI2). (**B**) Distribution of number of uniquely mapped PEs for each library. (**C**) Relationship between length of each transcript (bp) and depth of coverage of each transcript (number of mapped reads). (**D**) Shared and unique transcripts among aerial pod (P0), early subterranean pod (P1), seed (SD) and shell (SH) parts of subterranean pods. (**E**) Expression coverage versus total number of uniquely mapped PEs. A total of 226,587 transcripts (red line). Transcripts with FPKM > 1 are defined as expression (blue line). Expressed transcripts in seed (green line) and shell (orange line), respectively.

To better understand the molecular mechanism of peanut pod development, we attempted to perform the basic gene expression atlases regarding of pod development. We thought that the transcriptome landscape of peanut fruit was capable of building a theoretical foundation for future illustrating the molecular detail of hidden biological question under the appearance of pod growth. Therefore, 20 separated seed and shell samples representing 11 distinct stages (P0–P10) of pod development were sequenced with RNA-seq. Consequently, a total of approximately 2.87 × 10^8^ bp (approximately 62.45% of sequenced data) of uniquely mapped PEs (paired-end reads) were obtained for the subsequent abundance estimation **(**Fig. 1A). Of the uniquely mapped PEs, the number of reads mapped to different transcripts ranged from 1 to 1,705,381 with a median of 1,266 for the transcriptome. For individual libraries, such number ranged from 1 to 810,833, and the average number ranged from 82 for P0 to 208 for P4SH (Fig. 1B). The sequencing depth ranged from 0.24- to 175,758-folds with an average of 257-folds. The size of each transcript was plotted against the number of mapped reads on a logarithmic scale (Fig. 1C). The seed and shell tissues shared 51,264 transcripts with aerial pods (P0) and initial subterranean pods (P1) that are contained in shell and seed (Fig. 1D). The coverage analysis suggested that approximately 100 million short PEs should be sufficient to identify and measure all relevant transcripts during peanut pod development (Fig. 1E). A total of 226,587 transcripts were hit by more than 280 million PEs. Among expressed transcripts, 127,757 and 133,387 were expressed in seed and shell, respectively. In four scenarios, the number of transcripts that could be detected reached a plateau at approximately 100 million fragments.

### Dynamic reprogramming of pod developmental transcriptome

**Figure 2:**
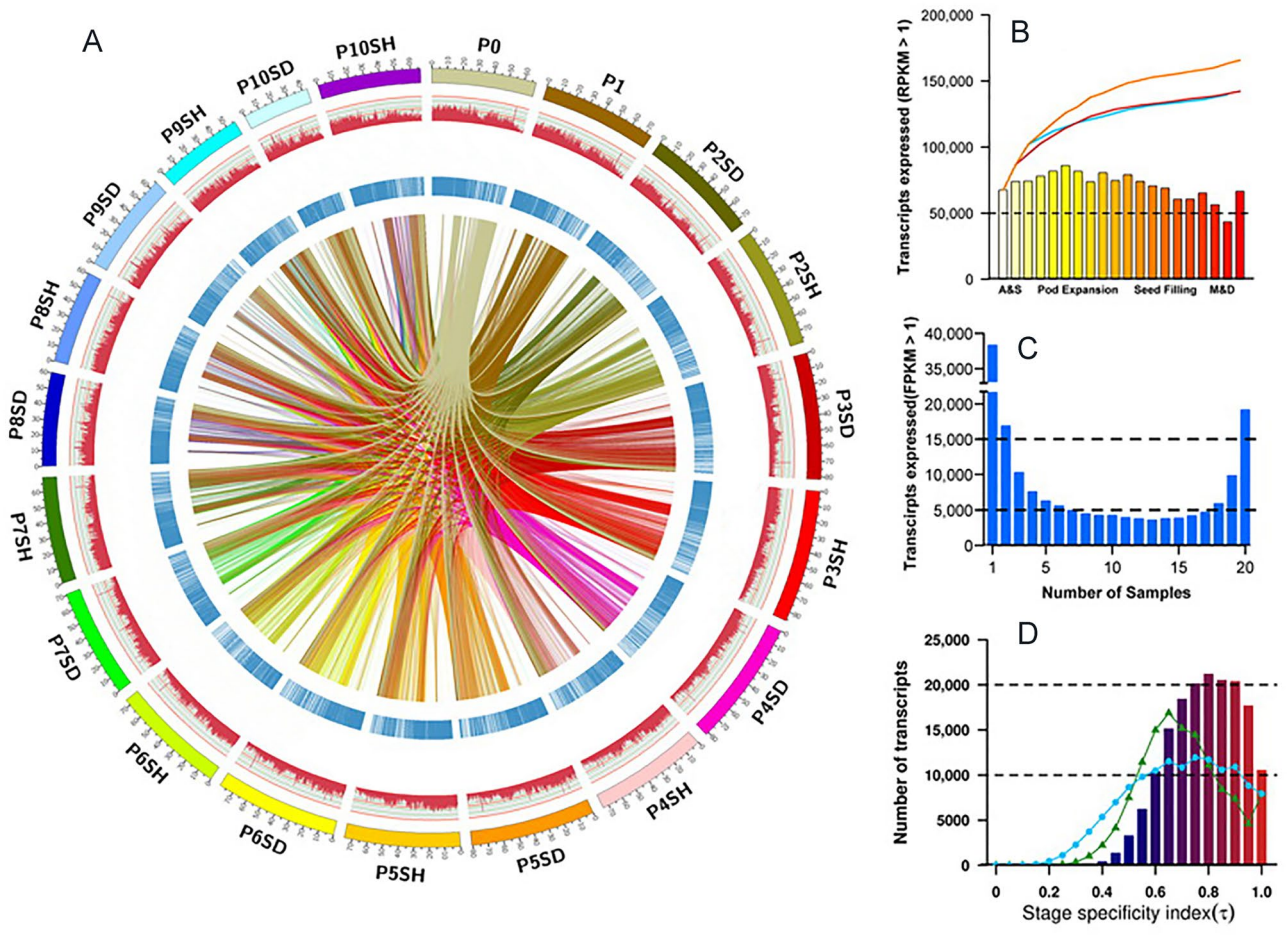
Active transcripts across peanut pod development. (**A**) Global transcriptional analysis of peanut pod development as determined by RNA-seq and circular visualization. The tick number (× 1,000) in the outer circle represents the number of expressed transcripts in each sample. The circular tracks are, going inwards: (1) FPKM data (≥ 10 and logarithm transformed) are represented in dark-red histograms. (2) The light blue track indicates expressed TFs in 20 individual samples. (3) Inner links represent shared transcripts between paired samples. (**B**) Expressed transcripts (FPKM > 1) across pod development. The bars indicate the number of transcripts expressed in each sample, and the lines indicate the cumulative number of expressed transcripts. The orange line indicates the cumulative number of expressed transcripts across all samples. The blue and brown lines indicate the cumulative number of expressed transcripts in seed and shell tissues, respectively. A&S, aerial and initial subterranean pods (P0 and P1); pod expansion, P2–P5; seed filling, P6–P9; M&D, mature and desiccation stages (P9 and P10). (**C**) Number of specific and shared transcripts expressed in 20 samples. (**D**) Distribution of *τ* values for 165,689 transcript profiles from all samples. The distributions of *τ* values are also shown for expressed transcripts from seeds (green triangle curve, 127,757 profiles) and shell (sky blue solid circle curve, 133,387 profiles). *τ* values varied between 0 for complete housekeeping genes with similar expression level in both tissues at all stages and 1 for strictly tissue-stage-specific genes.

The number of expressed transcripts (RPKM > 1) was similar in most samples with more than 50,000 transcripts, ranging from 34 to 52% of total transcripts detected in all samples (Fig. 2A, B, Table S3). One exception was the mature seed (P10SD), in which only 26% of transcripts were detected (Fig. 2B). The number of expressed transcripts was gradually increased, ranging from 67,567 (P0) to 86,178 (P3), and then it was gradually decreased to 43,483 (P10) (Fig. 2B). The number of transcripts expressed in single or multiple samples tended to shape a reverse parabolic distribution, in which the tissue-stage-specific, pair-shared and co-expressed transcripts constituted the largest group and accounted for 45% of expressed transcripts in pod (Fig. 2C). Although we detected ~ 20,000 co-expressed transcripts across all samples (Fig. 2C), specificity index (*τ*) analysis showed that none of transcripts were completely and constitutive expressed in both tissues across the entire development (Fig. 2D).

### Identification of DETs (differentially expressed transcripts) in developmental pod

**Figure 3:**
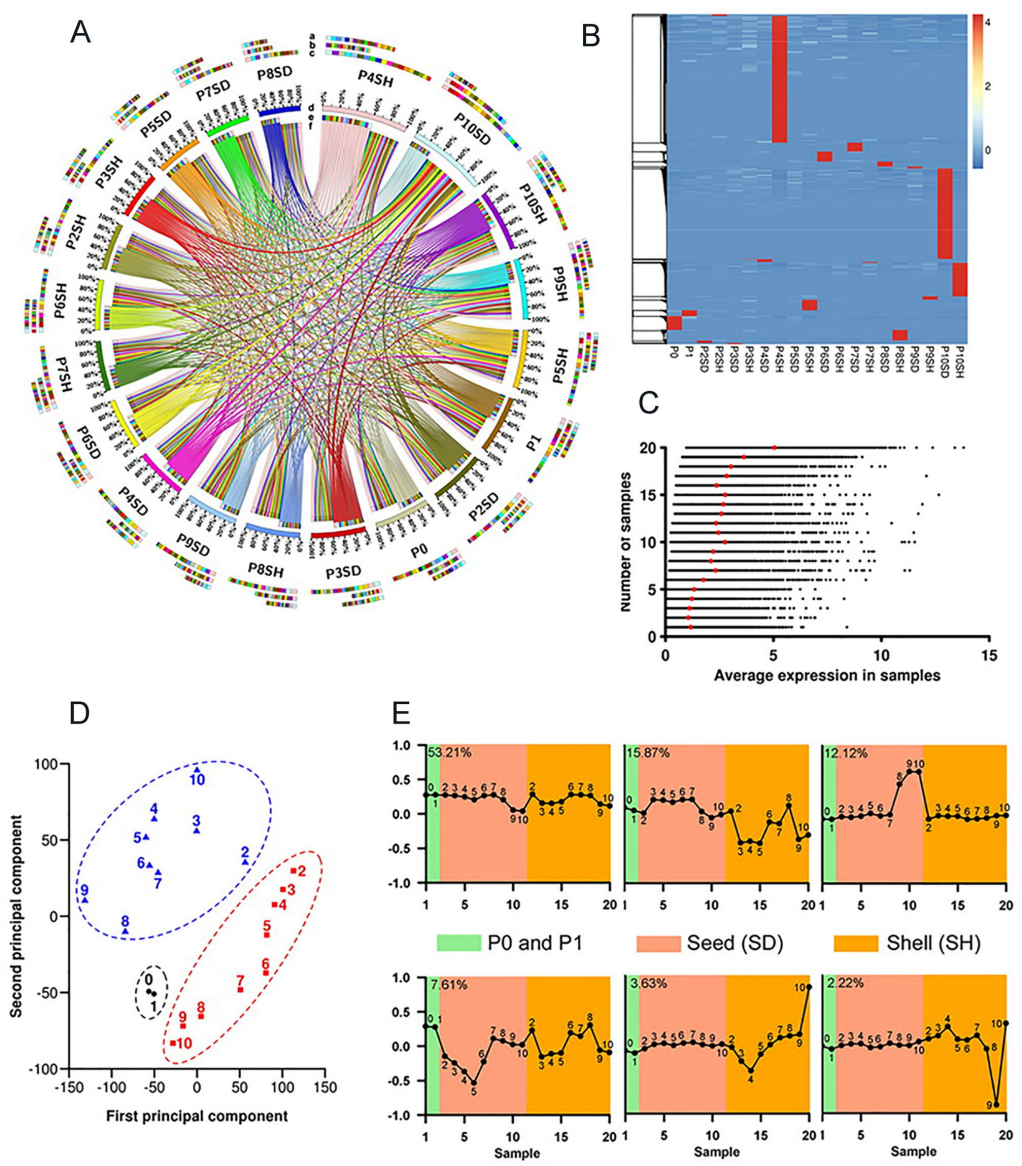
Expression dynamics during pod development. (**A**) DETs between libraries pairwise. The DETs were filtered using the three criteria as follows. (1) The FPKM of transcripts in the paired samples should be at least 1. (2) All DETs were identified by both DEGseq and GFOLD software programs. (3) The FPKM in one sample was at least twofold higher (up-regulation) or lower (down-regulation) than the FPKM in the other sample. Concentric circles were drawn to show DETs between paired samples using the Circos program. The ‘a’ and ‘e’ tracks represent up-regulated genes in the given sample compared with other samples. The ‘b’ and ‘f’ tracks represent down-regulated genes in the given sample compared with other samples. The ‘c’ and ‘d’ tracks represent all DETs (including up-regulated and down-regulated genes) in the given sample compared with other samples. Different colors in ‘a’, ‘b’ and ‘c’ tracks indicate the corresponding samples. The data-set used for this figure was supplied in Table S4. (**B**) Heatmap of tissue-stage-specific genes. Red, high expression; cyan, low expression. A list of these maker transcripts was provided in Table S5. (**C**) Average expression levels of transcripts expressed in one or multiple samples. (**D**) PCA was applied to 20 samples representing 11 developmental stages. Red squares represent seed, and light triangles represent shell. The two black solid circles represent P0 and P1. (**E**) PCA was applied to all expressed transcripts, and six trends were determined to together explain ~ 94% of the total expression variance across 20 samples. Contribution of each component is indicated on top left. Green represent P0 and P1 stages, Light brown presents seed, and stages are indicated by corresponding numbers (2–10). Orange represents shell, and stages are also indicated by numbers (2–10).

Despite the similar number of expressed transcripts in each sample, the underlying expression dynamics are greatly diverse during pod development. We identified 143,094 DETs in at least two samples (Fig. 3A, Table S4), representing 64% of the pod transcriptome. We identified a large number of tissue-stage-specific transcripts (FPKM > 5) in P4SH and P10SD, implying the more specialized nature for the tissues at these developmental stages (Fig. 3B, Table S5). We grouped transcripts based on the number of samples in which they were expressed. The average expression level was gradually increased from one sample to all samples (Fig. 3C). Relative expression levels in seed and shell tissues were both below the average levels, and those in shell were shifted towards lower values than those in seed. Despite the similar number of expressed transcripts and the large overlap across pod developmental stages, PCA (principal component analysis) found distinct transcriptional signatures in seed and shell tissues at various developmental stages (Fig. 3D). By applying PCA to all expressed transcripts, we identified six components that characterized ~ 95% of all expression trends (Fig. 3E).

### Dynamic repertoire of the shell transcriptome over pod expansion

**Figure 4:**
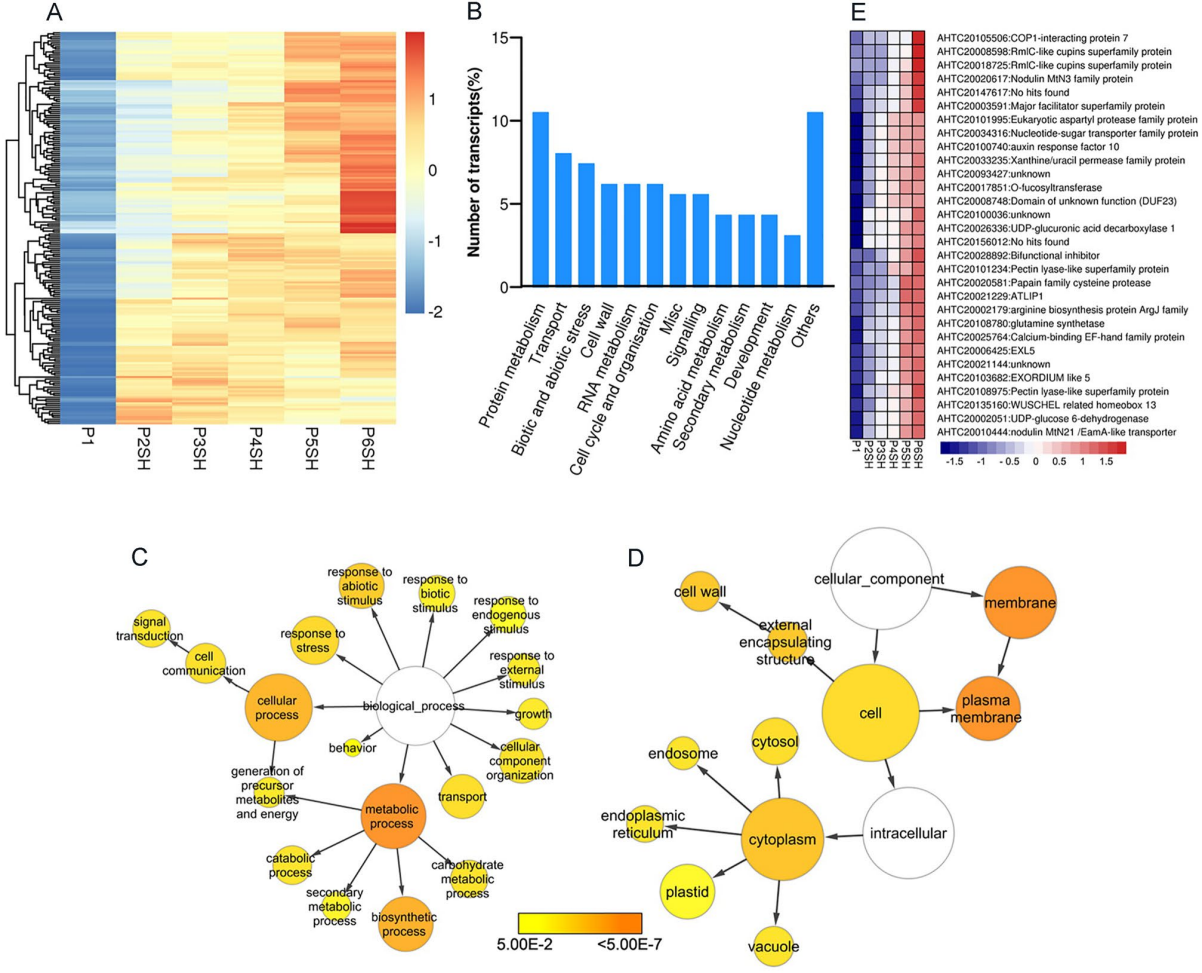
Coordinated changes of gene categories activated during pod expansion. (**A**) Heatmap showing expression profiles of up-regulated transcripts during pod expansion. (**B**) Functional distribution of transcripts in analysis of pod expansion. The distribution of transcripts among the 12 categories is shown. “Others” includes “not assigned” and eight minor categories. (**C**,**D**) Significantly enriched GO categories of up-regulated transcripts during pod expansion. The BinGO, a plugin for Cytoscape, was used to draw networks for the Biological Process and Cellular Component ontologies. Node size is proportional to the number of transcripts in each category, and the significance levels are color-coded ranging from 5E^−2^ to < 5E^−7^ (white, no significant difference; yellow, P = 0.05; orange, P < 5E^−7^). (**E**) Expression profiles of transcripts, of which the expression levels were monotonically increased during pod expansion. The transcript identifier and annotated function for each transcript were also shown.

Given that the period of expansion is related to shell, only P2SH to P6SH stages were considered. From our data-sets corresponding to the expansion period (P2SH to P6SH), we detected a total of 11,048 DETs between P1 stage and the expansion period (Table S6). Only 195 genes were activated during pod expansion, and their expression profiles were shown in Fig. 4A. Further software MapMan bins showed that most of these up-regulated transcripts across pod expansion stages encoded enzymes for protein metabolism, transport, stress, cell wall, RNA regulation of transcription, cell cycle and organization, signaling, and development categories (Fig. 4B, Table S7). GO analysis also revealed that they were associated with cell wall, membrane, signal transduction, and transport (Fig. 4C,D, Table S8).

Finally, we obtained only 30 transcripts, which were accompanied with pod enlarging (Fig. 4E, Table S9). KOBAS software mapped the 30 transcripts to 20 pathways, of which UDP-D-xylose biosynthesis pathway ranked the number one, followed by UDP-sugars inter-conversion. In agreement with enhanced cell wall synthesis, pod was gradually thickened, and its weight was increased during the pod expansion period (Table S10 and Fig. S1C), implying that UDP-D-xylose pathway played an important role in this period. Taking a broad view of expression levels of these transcripts during the entire pod development, we observed that the expressions of approximately 60% of these transcripts continued to increase in P7SH, and then decrease (Fig. S10, Table S11), indicating that these transcripts were expressed at high levels over pod expansion. At P6SH, our analysis showed that dynamic repertoire of the shell transcriptome was determined by the interaction of developmental and environmental factors. Peanut pod shell was not only characteristically viewed as a protective organ, but also played critical roles during the course of development.

### Developmental dynamics of the seed transcriptome

**Figure 5:**
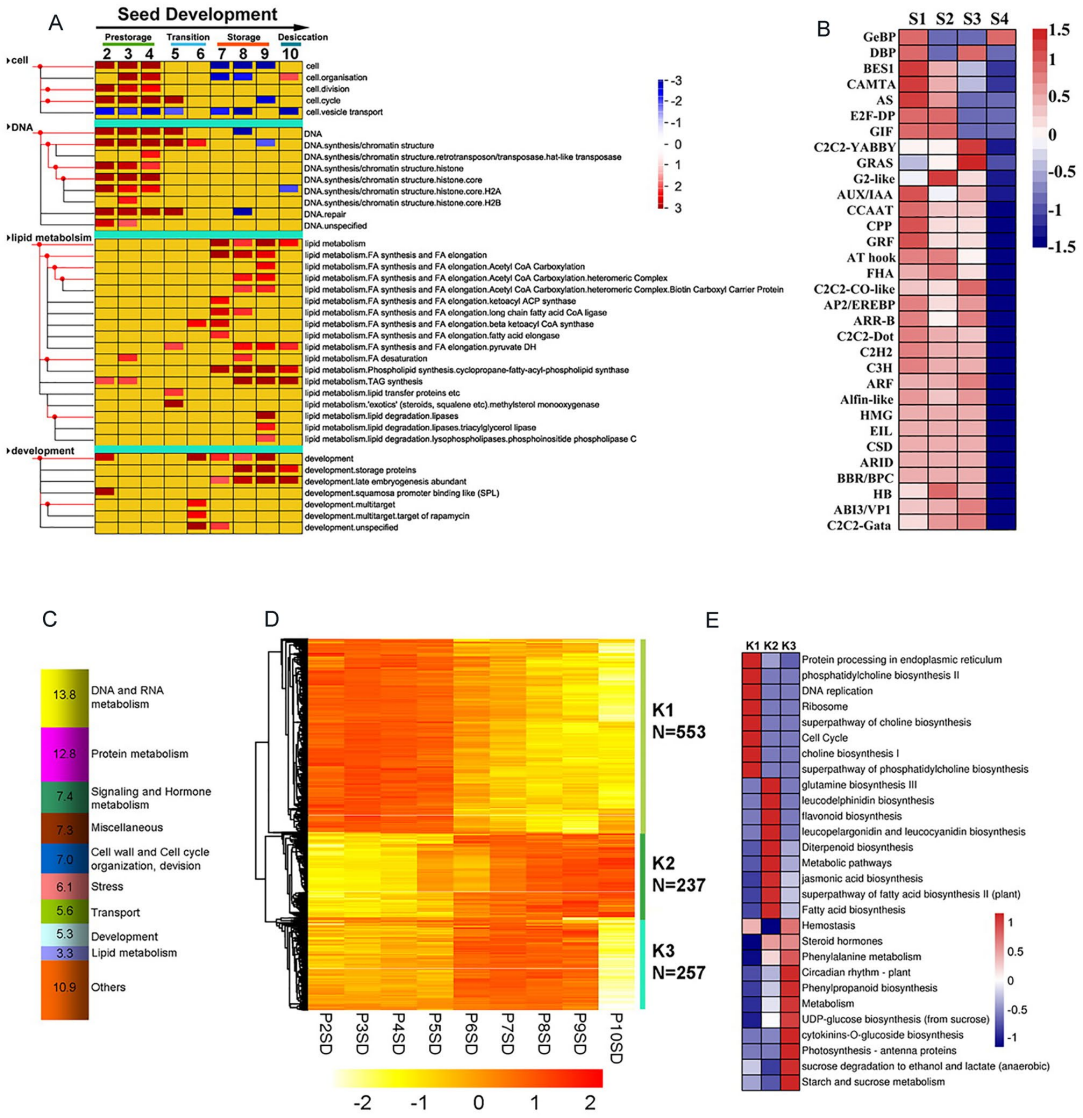
Dynamic development of the seed transcriptome during two major developmental periods. (**A**) PageMan display of changes of gene categories differentially activated along seed development. (**B**) Heatmap of 32 specific TF family during the seed development, S1(PSD2-4), S2(PSD5-6), S3(PSD7-9), S4(PSD10). (**C**) The distribution of the top nine functional categories of DETs is shown (excluding 17.2% belonging to ‘not assigned or unknown’). ‘Others’ includes 17 minor categories. (**D**) Heatmap showing expression profiles of DETs between period I (PSD2-5) and period II (PSD6-10). The number of transcripts (N) represented in each individual cluster is indicated on the right. (**E**) Pathways enrichment analysis among the three clusters using KOBAS 2.0. False colors are based on P values (P < 0.05). The full lists of pathways for the three clusters were provided in Tables S13–S15.

Firstly, PageMan^10^ software enrichment analysis showed that many biological activities were partitioned between the embryogenesis (P2SD to P5SD) and seed filling (P6SD to P10SD) (Fig. 5A). We detected 1,527 TFs during seed development. Among them, 96.5% of TFs were differentially expressed along the developmental gradient (Fig. S11). We also identified 32 family-specific-expression trends during seed development (Fig. 5B). Only few members of TF families were expressed during seed desiccation except for members of the GeBP (*Glabrous l* enhancer binding protein) family, which were involved in cytokinin responsed senesnence pathway. Additionally, DETs functional categories indicated that genes involved in DNA and RNA metabolisms accounted for 13.8% of annotated transcripts, followed by protein metabolism as well as signaling and hormone metabolism (Fig. 5C, Table S12). We identified three clusters (K1–K3) using hierarchical clustering analysis based on average-linkage method (Fig. 5D). Pathway enrichment analysis revealed that genes with high expression levels in the cluster K1 included protein metabolism, phosphatidycholine biosynthesis, and DNA replication (Fig. 5E, Table S13). Glutamine synthesis, diterpenoid biosynthesis, and fatty acid biosynthesis were greatly enriched in the cluster K2 (Fig. 5E, Table S14). Genes showed high expression levels during seed filling (P7SD–P9SD), but low expression levels during desiccation (P10SD), including circadian rhythm (plant), starch and sucrose metabolism, and hemostasis, suggesting that metabolic activities within the seed were dramatically down-regulated during desiccation (Fig. 5E, Table S15). Collectively, these results indicated that seed developmental dynamics were produced in part by highly dynamic, coordinated and periodic transitions in mRNA abundance.

### Photosynthetic genes are significantly up-regulated and enriched in aerial pod, and they play roles in pod development under dark conditions

**Figure 6:**
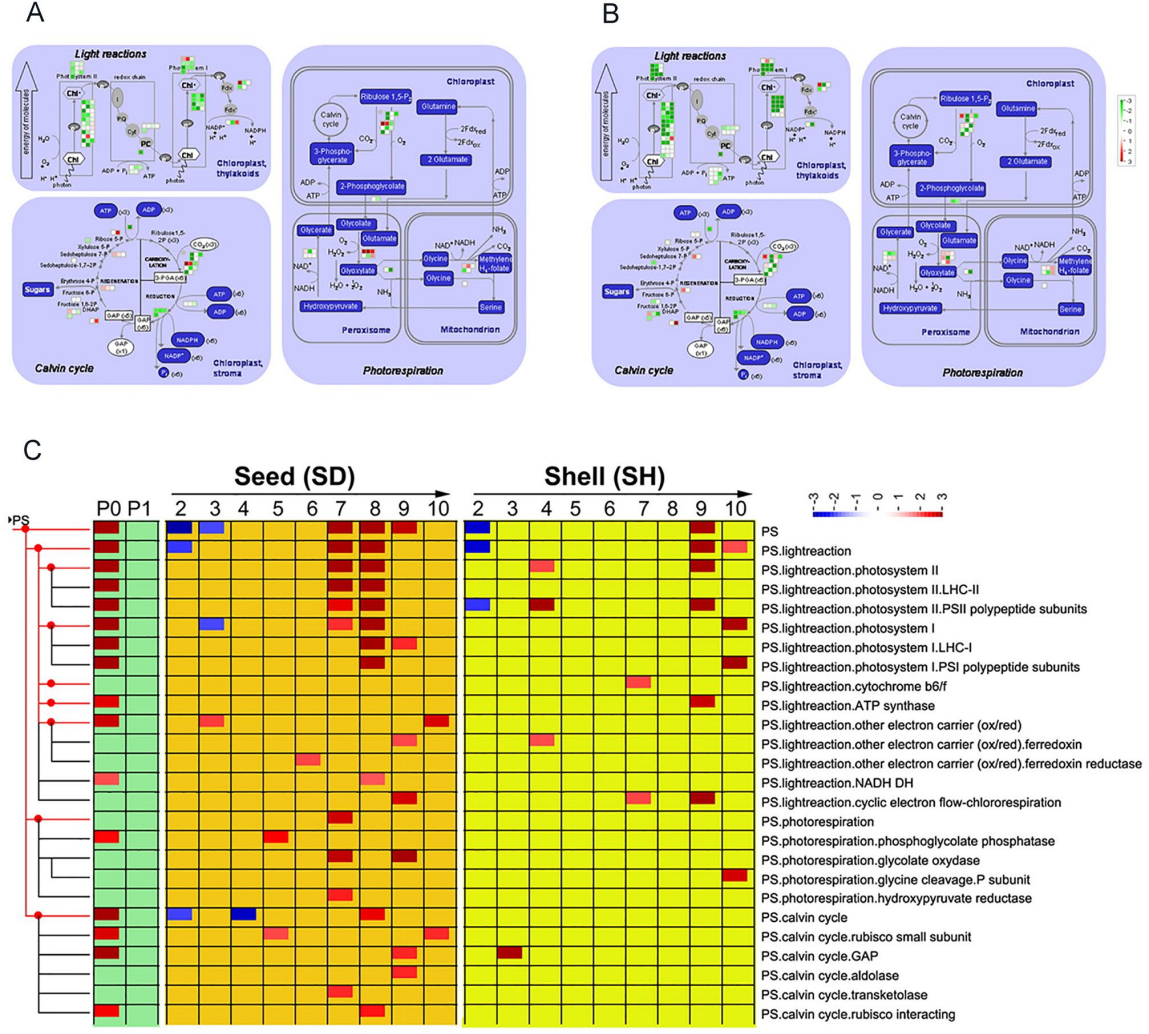
Photosynthesis involved in pod development. (**A**) MapMan photosynthesis overview maps showing differences at the transcript level between aerial pods (P0) and subterranean developing seeds. Log^2^ ratios for average transcript abundance in seed across stages P2SD to P10SD were calculated. (**B**) MapMan photosynthesis overview maps showing differences at the transcript level between aerial pods (P0) and subterranean shell tissue. (**C**) PageMan display of coordinated changes of photosynthesis during pod development. In (b) and (c), the logarithmic color scales from  − 3 to 3, green represents significantly higher expression in aerial pods compared with subterranean pods, and red represents significantly higher expression in subterranean pods compared with aerial pods. Red indicates significant enrichment of up-regulated transcripts; and blue indicates significant depletion of up-regulated transcripts.

Fruits from many species have been characterized to undergo a shift from partial photosynthesis to truly heterotrophic metabolism. A total of 211 transcripts identified to be involved in photosynthesis were analyzed for expression commonalities and differences in photosynthesis metabolic pathway during pod development (Table S16). We found pronounced expression changes in photosynthetic transcripts between aerial pod and subterranean pods, particularly in those of the Light Reactions (Fig. 6A,B). The majority of transcripts involved in the Light Reactions were down-regulated in subterranean pods, while many transcripts in subterranean pods were surprisingly up-regulated in the Photorespiration and Calvin Cycle. Many transcripts related to the Photosystem I and Photosystem II were up-regulated in P0 (Fig. 6C). Strikingly, up-regulated transcripts related to photosynthesis were also significantly enriched in three consecutive developmental points (P7SD, P8SD and P9SD). Additionally, during seed maturation (P9SH and P10SH), transcripts related to photosynthesis were also significantly up-regulated. Moreover, qRT-PCR was carried out to validate the relative expressions of photosynthesis transcripts at five representative stages (P0, P1, P2, P7 and P10), of which 17 highly expressed transcripts in aerial peg presented the consistent trends with RNA-seq result during seed formation (Fig. S12). Taken together, transcripts related to photosynthesis were obviously enriched in aerial pods relative to subterranean pods, while these transcripts also were up-regulated at various developmental stages underground, implying that photosynthetic genes probably played potential roles in subterranean pod development under dark condition.

## Discussion

As the main harvesting organ, peanut fruit develops underground, and it is undisputedly an important organ from agronomic and biological perspectives. Currently, we conducted histological surveys at 1-mm intervals and deep transcriptomic surveys for one stage aboveground and 10 developmental stages underground. We mapped the transcriptional changes along with pod development. Based on 20 samples representing 11 distinct stages, we progressed toward a broad understanding of the dynamic changes in the transcriptome during peanut aerial peg formation to subterranean pod, including pod expansion together with embryogenesis, seed filling, and desiccation. Here, the number of expressed transcripts was more than 50,000 in most samples. The only exception was the mature seed (P10SD), in which approximately 43,000 transcripts were detected. Actually, this result indicated that plant decreased the number of seed transcripts when the seed began to prepare for dormancy^11^, and several transcripts related to flavonoid pathway were identified to probably modulate the pod dormancy and seed germination (Table S26). However, little information is avaliable with respect to the number of protein-encoding genes and transcripts derived from alternative splicing in the process of peanut pod development. This aggravates the existing difficult task in estimating how many transcripts are expressed in peanut pod. Fortunately, high-throughput RNA-seq can relatively detect rarely expressed transcripts due to the high coverage. On the other hand, peanut pod under growth condition fully filled with darkness and soil induced mechanical pressure, pod shell expanded early than seed development, and shell had to evolve with ability to suit for subterranean circumstance as a protective and perceive organ to guarantee subsequent seed swelling. Therefore, peanut fruit growth condition with many complex features directly restricted the molecular mechanism mining in pod. Transcriptome data provide an opportunity for identifying critical growth pathway during peanut fruit formation, which especially facilitate graphic interpretation correlated with several modules, such as UDP-glucose deydrogenase and cell wall synthesis pathway in shell, fatty acid biosynthesis (Fig. S13 and Table S26) and photosynthesis in seed. Taken together, our results cataloged gene expression patterns across the aerial and underground in developing pod, extended our knowledge on pod growth in darkness, analyzed the expression patterns to define groups of annotated, co-regulated preferentially expressed genes at different time-points, and characterized the major development processes, like pod expansion, seed filling and desiccation. Our data could serve as a valuable resource of peanut pod transcripome study for developmental biologists who are interested in demonstrating fruit development mechanism under dark conditions, as well as for the general transcriptomics community.

### Peanut fruit photosynthesis is seemingly important in aerial pod and also plays a paramount role in seed development

We observed that 785 transcripts were preferentially expressed in aerial pod (Table S17). Half of them have been also identified as aerial-pod-preferred genes in our previous study^3^. The remaining 50% of transcripts might be due to material collection and sequencing coverage. Previously, aerial pods have been treated to prevent the pods from going into soils, and samples were pooled from five time points, while we collected aerial pod at only at one time point in this study. Additionally, we found a 60-folds sequencing coverage for P0 (aerial peg) in this study, while approximately five-folds sequencing coverage is found for aerial pod in the previous study. Consistent with the previous study, photosynthetic genes were up-regulated and enriched in aerial pod in this study. Moreover, we observed that photosynthetic genes also played roles in seed development. A recent study has shown that fruit photosynthesis is not necessary for energy metabolism or development, but it plays a role in timed seed development^12^. Furthermore, we found that photosynthetic genes showed enrichment of up-regulated genes not only in aerial pod, but also in P7 through P9 (Fig. 6C). The expressions of genes encoding the biochemical reactions of the photorespiratory cycle and calvin cycle were mostly up-regulated. Our results indicated that photosynthesis had effect on aerial pod and late pod development in the seed and shell (Fig. 6C), while the biological reason was still unclear due to lack of sufficient study reference. Therefore, a further interpretation is able to portend the intellectual extension of plant fruit development under dark condition.

### Distinctive skotomorphogenesis in peanut pod development underground

Light is one of the most important factors modulating many developmental processes of plants^13,14^. Light represses peanut pod swelling and embryonic development in aerial pod. Pegs from underground flowers lack the light-induced inhibition to fruit enlargement. When the peg starts off already underground, there is no pause in embryo growth^15^. The ubiquitin ligase COP1 (constitutively photomorphogenic 1) negatively regulates plant photomophogenesis^16^. We found that COP1 gene was co-expressed across pod development, and the COP1 interacting protein 7 (CIP7) has been reported to be up-regulated by light^5^. Previous study on peanut gynophore development in darkness has reported that the expression of CIP7 is drastically decreased in dark-grown gynophores^17^. The study only covers three developmental stages, not including the following pod development. However, we found that the expression of CIP7 was decreased when gynophores just penetrated into soil, and then it was monotonically increased during pod expansion underground. Moreover, other genes involved in light signaling transduction, such as PHYA (phytochrome A), PHABULOSA B, PHABULOSA C, CRY2 (cryptochromes 2) and CRY3 (cryptochromes 3), were also lowly expressed during pod development (Table S16). However, it is necessary to further investigate the roles of these genes in peanut fruit development in the absence of light.

### Seed development can be divided into two lag phases in peanut, like other legumes

According to phenotypic analysis, we could coarsely divide the seed development into two developmental periods. In the first period (from P2SD to P5SD), the size of peanut seed varied a little (~ 2 mm). By contrast, the size of peanut was increased to approximately 12 mm in the second period (from P6SD to P10SD), approximately six-folds larger than the initial size (Table S1). Strikingly, the two periods could be distinctly separated by gene expression (Fig. 5D). We found that the first period was enriched in activities for cellular functions, such as cell cycle regulation, cell division and DNA synthesis, while the second period depleted, suggesting that cells were increased in number in the first period, but increased in size in the second period. This finding was consistent with previous studies in pea seed development^18^. Studies of pea have identified three rapid phases of seed growth separated by two lag phases. In the first lag phase, the embryo mainly grows by cell division, and the second one is characterized by cell expansion^19^. According to gene expression, the second period could be subdivided into two periods, including mature (P7SD–P9SD) and desiccation (P10SD). During the seed filling period, there was the enrichment of up-regulated genes in transport functional categories for both seed and shell since this was the period for transition from sink (shell) to source (seed) tissue. Additionally, 78 fatty acid genes and 104 genes related to TAG (triacylglycerol) metabolism were identified during seed filling (Fig. S13 and Table S26), while their transcriptional patterns were still unclear along with seed maturation. Actually, *AHTC20004627*, a homologous of *Arabidopsis* AFL (ABI3/FUC3/LEC2) complex, encoding the ABI3/VP1-related B3-domain-containing TF was up-regulated in ripe seed, which probably contributed to the elevated transcript abundance of oil synthesis gene, and further demonstrating their relationship will offer a valuable gene resource to improve the quality of peanut oil.

Collectively, pod is a key component to peanut yield, even though we have harvested many advance progresses in peanut breeding practice, but some particular biological question regarding of peanut pod development still need to be explored deeply. Here, through generating a highly resolved and extensive transcriptome map, we set up a solid framework for a systemic approach to understand peanut pod development underground. This understanding also provides deeper insights on multiple fields of pod biology under dark condition, including TF regulation, phytohormone signaling transduction, and process of photo synthesis. Meanwhile, our finding appreciably expanded the number of building blocks used to make a peanut fruit at the transcriptional and post-transcriptional levels. For future, comprehensively deciphering and integrating the transcriptome data-set is capable of assisting breeder and biologist to understand the regulatory events of pod formation and thus determined yield in peanut.

## Methods

### Plant materials and RNA isolation

Plants of “Hanghua 2hao”, a widespread peanut cultivar in southern China, were grown in fields of the experimental station of Guangdong Academy of Agricultural Sciences. Aerial and subterranean pods were collected from plants grown in the field. Selfed flowers were identified with colored plastic thread, and elongating aerial pegs were tied with colored tags on the 8th day after flowering (DAF). A total of 20 samples were prepared in this study. These samples were obtained from one aerial and 10 subterranean stages. The stages were mainly defined by pod diameter and seed diameter using DAF as reference. The pod diameter was determined at the base of the pod adjacent to the gynophores, and the seed located at the base of the pod was used to measure the diameter. The fruits were classified based on size rather than time from flowering because it was more reproducible under different growing conditions. The detailed information for sample collection was provided in Table S1. Light microscope observation was displayed in Fig. S1 as previously described^3^. Total RNA was extracted from all tissue samples using the RNeasy Plant Mini Kit (Qiagen). The quality and quantity of each RNA sample were analyzed using NanoDrop (Thermo Scientific) and the Agilent 2100 Bioanalyzer (Agilent), respectively. For reducing the cost, equal quantities of total RNA from three biological replicates were pooled for mRNA purification, followed by library preparation for each sample.

### Illumina sequencing and assembly

Briefly, mRNA sequencing was performed at MacroGen Inc. (https://www.macrogen.com) using the Illumina HiSeq2000 platform. Library construction and sequencing were carried out following the standard sequencing protocols recommended by Illumina. DNA fragments for each library were listed in Table S2. Data processing of mRNA sequencing and assembly were described in Fig. S2 and Appendix A: Supplementary Data 1.

### RNA-seq data alignment

SSAHA^20^ was used to map paired-end reads (PEs) to the reference transcriptome (AHGI2)^21^, allowing five mismatches at most. Reads with multiple matches were removed from the primary search results. For each pair of forward and reverse reads, both ends were required to uniquely map to the same transcript. After these filtrations, a set of uniquely mapped pairs were collected for the subsequent abundance estimation. Using the uniquely mapped read pairs, the expression levels of transcripts were estimated with Fragments Per Kilobase of transcript per Million mapped fragments (FPKM)^22^ in a way similar to reads per kilobase of exons per million mapped reads (RPKM)^23^.

### Assessment of gene expression

In the present study, two programs were employed to identify differentially expressed transcripts (DETs) (Transcripts that were defined as DETs by both programs were considered to be DETs). First, DETs were determined from different samples using an R package (DEGseq) proposed by a previous study^24^. For each gene, the P-value and Q-value were calculated. Then, the significant threshold to control the FDR at a given value was computed. Subsequently, DETs were identified using the GFOLD package^25^. Reliable statistics was assigned by GFOLD for expression changes based on the posterior distribution of log fold change. Moreover, an in-house Perl script was used to extract DETs from the output files generated by both programs. Any DETs detected by only one program were ignored and not used for further analysis.

The expression values for all samples were also converted into Z scores using a tow-step process as described in a previous study^26^. To facilitate graphical interpretation of tissue and stage relatedness, principal component analysis (PCA) was carried out to detect the major source of expression variances underlying development using R.

### Tissue and stage specificity of gene expression

Tissue and stage specificity of gene expression during pod development was measured using a single statistical analysis by *τ* value for tissue specificity index:

$$\tau_{i} = \sum\limits_{i = 1}^{N} {(1 - R_{i,j} /R_{i,\max } )/(N - 1)}$$

where N is the number of samples, R (i, j) is the expression value of i gene in j sample, and R (i, max) is the maximal value of gene i in all samples surveyed.

### Functional annotation and analysis

Transcripts were subjected to BLASTX analysis against the following databases: Uniprot Viridiplantae database for deducing putative function; UniProt *Arabidopsis* dataset for KOBAS^27^ analysis,and the *Arabidopsis* protein database (TAIR) for MapMan^28^ mapping. An E-value threshold of 1E^−5^ was used to determine the significant hits. The putative functions of query transcripts were defined by the first subject hits. An in-house Perl script was used to perform gene ontology (GO) annotation based on UniProtKB GOA file (ftp://ftp.ebi.ac.uk). KOBAS (KEGG Orthology Based Annotation System, v2.0) was used to identify biochemical pathways and calculate the statistical significance of each pathway. The UniProtKB accession numbers assigned to peanut transcripts were submitted to KOBAS for searching known pathways in the KEGG database.

### qRT-PCR analysis

To validate the RNA-seq results, qRT-PCR was conducted as previously described^29^. All assays for a particular gene were performed in triplicate synchronously under identical conditions. The *Ah18S* gene was used as an internal reference. Relative expressions of all target genes were calculated using the 2 ^–ΔΔCT^ method. The relative expression values were then validated for the RNA-seq data.

## Supplementary information

Supplementary information.

Supplementary figures.

Supplementary tables.

## Abbreviations

P0: Aerial peg
P1: Subterranean peg
PSD: Pod seed
PSH: Pod shell
PEs: Paired-end reads
P2–P5: Pod expansion
P6–P9: Seed filling
P9–P10: Mature and desiccation stages
RPKM: Reads per kilobase per million mapped reads
FPKM: Fragments per kilobase million
TFs: Transcription factors
A&S: Aerial and initial subterranean pods
DETs: Differentially expressed transcripts
PCA: Principal components analysis
GO: Gene ontology
KEGG: Kyoto encyclopedia of genes and genomes
GeBP: Glabrous l enhancer binding protein
COP1: Constitutively photomorphogenic 1
CIP: COP1 interacting protein
PHYA: Phytochrome A
PH: Phabulosa
CRY: Cryptochromes
TAG: Triacylglycerol
