# Supplementary material for: Global transcriptome analysis of subterranean pod and seed in peanut (Arachis hypogaea L.) unravels the complexity of fruit development under dark condition
Source: Sci Rep. 2020 Aug 3;10:13050. doi: 10.1038/s41598-020-69943-7 (PMC7398922; doi:10.1038/s41598-020-69943-7)
Supplement: Supplementary file 1 — Supplementary information. [file 41598_2020_69943_MOESM1_ESM.pdf]

Global transcriptome analysis of subterranean pod and seed in peanut (*Arachis hypogaea* L.) unravels the complexity of fruit development under dark condition

Hao Liu[1], Xuanqiang Liang[1], Qing Lu[1], Haifen Li[1], Haiyan Liu[1], Shaoxiong Li[1], Rajeev Varshney[2], Yanbin Hong[1*], Xiaoping Chen[1*]

[1]Guangdong Provincial Key Laboratory of Crop Genetic Improvement, Crops Research Institute, Guangdong Academy of Agricultural Sciences (GAAS), Guangzhou 510640, China.

[2]International Crops Research Institute for the Semi-Arid Tropics (ICRISAT), Patancheru 502324, India.

* Corresponding. chenxiaoping@gdaas.cn, hongyanbin@gdaas.cn.

Appendix A. Supplementary Data Note

1. Illumina sequencing

In this study, 20 paired-end (PE) cDNA libraries of ~500 bp were constructed for transcriptome sequencing using RNAs from peanut pods representing 11 developmental stages (**Table S1 and Fig. S1**). The library construction and sequencing were performed on Illumina HiSeq2000 platform at MacroGene Inc. (www.macrogene.com) following the manufacturer's protocols (Illumina, San Diego, CA). In total, 1 004 103 564 sequence reads were generated, encompassing about 101 Gb of sequence data in fastq format (**Table S2**).

2. Preparation for assemble peanut transcriptome

All the sequence cleaning and assemblies were carried out on a server with 16 cores and 64 Gb random access memory. We employed a combined assembly strategy[1, 2] to yield the comprehensive peanut reference transcriptome (**Fig. S2**). First, we used long ESTs (Sanger and 454) to construct an initial reference transcriptome. Second, we mapped HiSeq2000 reads to the initial reference and obtained the unaligned reads. Third, we assembled unaligned reads and singletons using TGICL2.0 (TGI Clustering Tools)[3], generating new contigs. Finally, we pool new contigs and the initial reference to come up with the comprehensive peanut reference transcriptome with duplicates removed.

First, long ESTs (Sanger and 454) were assembled using TGICL2.0 program to generate contigs as an initial reference transcriptome. Second, HiSeq2000 reads generated in this study were mapped to the initial reference and the mapped and unaligned reads were separated. Third, the unaligned reads were assembled using oases with an optimized k value (k = 27), generating transcripts derived-from short reads. And singletons from long ESTs and processed contigs assembled from short reads were then used together for a super assembly using TGICL. Finally, we pool new contigs and the initial reference to come up with the comprehensive peanut reference transcriptome with duplicates and isoforms removed.

The raw short reads were trimmed and filtered for vector and contamination using Lucy1.20p[4, 5]. A total of 955, 043, 570 reads passed the read clipping and filtering (the raw and filtered reads shown in **Table S3**). The Sanger ESTs used for assembly were from GenBank dbEST (www.ncbi.nlm.nih.gov/dbEST/index.html) and generated in house. The 454 reads were from GenBank SRA database (www.ncbi.nlm.nih.gov/sra/) (GenBank accession numbers: SSR042413, SSR042414, SRR042415, SRR042416, SRR042421, SRR042422, SRR057708, SRR057709, SRR057710, SRR057711, SRR057712, SRR057713, SRR057714, SRR057715, SRR057716, SRR057717, SRR057718, SRR057719, SRR057720, SRR057721, SRR057722, SRR057723, SRR057724, SRX149735, SRX149736, SRX149737). In total, 4, 619, 956 raw ESTs were collected and filtered using

SeqTrim (-vp 30 –nr -0.05) against UniVec build #7 (http://www.ncbi.nlm.nih.gov/VecScreen/UniVec.html). The summary of all long source ESTs is shown in Supplementary Table 5. After removed ESTs less than 100 bp, this resulted in a total of 3, 670, 358 high-quality ESTs, which were used for assembly using TGICL2.0[3]. After assembly, 103, 439 contigs and 675, 904 singletons were generated (**Fig. S2**). The contigs were used as an initial reference for short reads mapping in the subsequent assembly.

Because identical reads do not contribute to mapping, consolidation of identical reads into a single representative sequence may reduce the computational resource requirements. Duplicate reads were removed using CD-HIT-EST[6] with parameters –c 1, -aS 1. A total of 129,031,193 unique high-quality reads were obtained and mapped onto the initial reference. The mapped reads were not taken into account for assembly. The remaining 48,588,882 unaligned reads were used for assembly. The choice of best assembler for assembly relies on the data set and needs to be optimized[7]. To obtain the best assembly results with the unaligned reads, we used data set from P0 to optimize the assembly using three assembly programs including Trinity[8], SOAPdenovo (v1.04, up to 127 kmer, http://soap.genomics.org.cn) and Oases (v0.2.08)[9]. Three data sets were used as input in all three programs. The three data sets include raw sequence reads (Raw), filtered reads (Filtered) and filtered reads with duplicate removed (NoDup). Oases and SOAPdenovo assemblies were performed at various *k*-mer lengths. Trinity assembly of the short reads was performed using default parameter on 16 CPUs (--CPU 16) with 20 Gb heap space (--bflyHeapSpace 20G). We assessed the effect of various *k*-mer lengths through comparison of N50, average length, number of used reads and number of contigs (**Fig. S3**). Trinity used the three data sets as input and generated assemblies with N50 less than 500 bp and average lengths of 265-322 bp. Taken together, Oases with k=27 was used for assembly of unaligned reads and generated 361,346 contigs (**Fig. S2**). All singleton ESTs from TGICL using Sanger and 454 ESTs as input and contigs assembled from HiSeq2000 reads using Oases (k=27) were used for a super assembly using TGICL, generating 258,669 contigs. Combining the initial reference

and assembly, a total 352,108 contigs were obtained through the combined assembly strategy. We used CD-HIT-EST to remove redundancy and isoforms and retain the longest possible contigs. Finally, we produced a new peanut reference transcriptome named AHGI2, consisting of 287, 580 transcript assembly contigs with more than 100 bp in length and representing approximately 193 Mb of transcript sequence and 6.89% of the peanut genome (2,800 Mb). We also compared the new assembled reference transcriptome with our previous reported reference transcriptome AHGI1[10], NCBI Peanut UniGene Build #3 and PeanutDB (http://bioinfolab.muohio.edu/txid3818v1/) (**Table S19**).

3. Strategy for sequencing of mRNA during pod development as well as assembly of the peanut transcriptome

To analyze the functional complexity of the peanut pod transcriptome and comprehensively characterize their transcriptional dynamics along development, as well as discover potential molecular mechanism underlying development underground, we conducted shotgun sequencing of mRNA[11] to generate randomly distributed reads at high resolution. Total RNA was isolated from 20 separated seed and shell samples representing 11 distinct stages of pod development (**Table S1**). Developing embryos along development have also been analyzed in great detail at the light microscope level (**Fig. S1**). The 20 samples comprised one aerial stage and 10 subterranean stages. All samples were run as individual samples, producing 101-bp paired-end reads for mRNA on the Illumina HiSeq2000 platform (**Table S2**). In total, we generated more than one billion reads for RNA-seq, equal to 101,414,459,964 bp of transcripts, representing over 36-fold coverage of the peanut genome (**Table S18**).

Assembly of the peanut transcriptome was optimized with a combined assembly strategy (**Fig. S2 and S3**). After a step-by-step optimization of the assembly of the transcriptome, we report a total of 287,580 transcript assembly contigs in peanut representing 193,141,170 (193 Mb) of the sequencing with an N50 of 1003 bp and an average contig length of 671 bp. For convenience and clarity, hereafter,

‘transcript’ refers to transcript assembled contig. A unique transcript identifier (ID) number has been assigned to all transcripts from AHTC20000001 to AHTC20287580. Among these transcripts, 33,859 were novel transcripts detected in this study. GC content of peanut transcripts was determined and compared with other legumes (soybean, *Medicago. truncatula*, *Lotus. japonicus*, chickpea), dicot (*Arabidopsis*) and monocot (rice and maize) species (**Fig. S4**). The GC content distribution of peanut transcripts is bimodal. Similar observations were found for the transcripts of other legume species with the exception of *L. japonicus*. The range of GC content was narrower in legume species compared to monocot species (rice and maize).

4. Analysis process of transcriptome landscape of peanut pod development

To estimate how many genes were expressed and to profile the transcriptome at different developmental stages comprehensively, we aligned every individual library’s data against the reference transcriptome sequence using SSAHA 2[12]. Tolerances were set to allow up to five mismatches in each alignment, and reads that aligned to multiple reference transcripts were ignored. By these criteria, 50.54%-66.17% of paired-end reads (PEs) were uniquely mapped to the reference transcriptome and 1.70%-2.79% of PEs was filtered as reads with bad matches (**Table S20**). The remaining reads (31.95%-47.12%) were defined as unmapped reads. Furthermore, a trend of an increase in the length of the transcript resulting in an increase in the number of mapped reads was not observed with a low R2 value (R2 = 0.2006). Using the uniquely mapped PEs, we estimated the expression levels of transcripts using fragments per kilobase of transcript per million mapped fragments (FPKM)[13]. To circumvent background noise of the experiment and exactly estimate how many transcripts were expressed throughout the pod, a total of 165,689 transcripts with FPKM $\geq$ 1 were defined as expressed transcripts. Among them, 127,757 and 133, 387 were expressed in seed and shell, respectively.

5. Additional illustration of dynamic reprogramming of pod developmental transcriptome

We examined the gene expression dynamics throughout pod development using RNA-Seq data. We detected ~40,000 transcripts which are tissue-stage-specific transcripts expressed in only one sample. A diversity overlaps in transcripts (from 35.6% to 73.4% between pairwise samples, averaging 55.8%, s.d.=7.8%) are present in distinct stages and tissues, although a similar number of transcripts is detected in each stage. It is worth mentioning that P10SD shows the lowest overlap with others samples, averaging 38.7% (s.d.=2.6%), suggesting a different transcriptional program present at this stage **(Table S21)**. Right shift of the peak of τ values revealed that expression of most transcripts should be tissue- or stage-specific during pod development. Pearson correlation coefficients also indicated that diverse expression occurred during pod development. Correlation coefficients between different samples ranged from 0.0258 for P1 and P10SD to 0.9762 for P3SD and P4SD (**Fig. S5-a**). Most of stages are highly correlated with their adjacent stages. Pearson correlation coefficients between two consecutive stages within seed tissue ranged from 0.6517 to 0.9762 (**Fig. S5-b**), indicating excellent concordance with each other. Nevertheless, correlation coefficients between two consecutive stages within shell tissues ranged from 0.2724 to 0.9520 (**Fig. S5-c**). In addition, the number of identified transcription factor transcripts (TFs) was also similar in most samples (**Fig. 3-A**), ranging from 41% to 48% of TF genes from *Arabidopsis* (TAIR database). Exceptions are P9SH and P10SD, in which only 36% and 32% of TFs were identified, respectively. Notably, seed and shell shared most of TFs at the same stage (**Fig. S6**). Strikingly, down-regulated transcripts in P10SD accounts for ~80% of differentially expressed (DE) transcripts when compared with other samples. We employed qRT-PCR to validate the expression levels of 30 randomly selected transcripts and observed a high correlation (R^2=0.9545, **Fig. S7**). To understand the dynamics of gene expression over the developmental time course we grouped transcripts using the K-Means clustering algorithm. We identified 20 clusters

representing a variety of developmental gene expression patterns (**Fig. S8 and Table S22**). Consistent with differential expression analysis, the number of specific transcripts varies greatly in samples. Only a few specific transcripts were found in P2SH, P3SD, P3SH, P5SD and P7SH, perhaps reflecting different expression dynamics. Genes that are detected in few stages are expressed at low levels, while genes that are detectable in most stage are expressed at high levels. Expressed transcripts were marked by a negative offset distribution below average levels, with few expressed above average levels (**Fig. S9**).

To facilitate graphic interpretation of relatedness of samples or developmental stages, we analyze the data using principal component analysis (PCA). In seed and shell clusters, we could identify three subgroups differentiated by developmental stages within either cluster. Therefore, the first two components can probably be attributed to transcriptional changes in the tissues across the pod developmental stages. PCA showed a clear influence of the explaining variance (PC1=53.21%, PC1+2=69.08%, PC1+2+3=81.2%, PC1+2+3+4=88.81%) (**Fig. 3-D and 3-E**). These components highlight differences between seed and shell and between different developmental stages. The last two stages (P9 and P10) showed dominant differences in both seed and shell tissues. Within the second component, seed and shell stood out with opposing component. This component may probably be attributed to the tissue difference, implying the distinctiveness of seed and shell that contribute approximately 15% of total expression variance.

6. Additional illustration for Dynamic repertoire of the shell transcriptome over pod expansion

Here, we attempt to focus on what happen to peanut pod following penetration into the soil. According to material collection information, pod expansion started from P2 and then reached the largest diameter (~16.5 mm) at the P6 stage when the seed size is ~ 2 mm and seed development is switching from late embryogenesis to seed filling. Given that the period of expansion is relate to shell, only P2SH to P6SH stages are considered. From our data sets corresponding to the expansion period

(P2SH to P6SH), we detected a total of 11,048 DE transcripts between P1 stage and the expansion period (**Table S6**). To confidently identify a set of transcripts whose transcription is activated during pod expansion, we imposed three criteria. First, we required an average FPKM of at least 1 for each transcript. Second, we require that the FPKM at P2SH to P6SH is at least twofold higher than the FPKM at P1 stage. A total of 3,291 genes passed the two filters (**Table S23**). Third, we require the expression level from P2SH to P6SH is increasing, allowing for a decrease by 1 FPKM or a 10% drop in expression level between two consecutive developmental stages. We then used MapMan[14] annotation to assign transcripts to functional categories. Strikingly, GO analysis of these genes showed that a broad array of stimuli, including abiotic and biotic, external and endogenous stimulus, were assigned to these up-regulated transcripts (**Fig. 6-C**), revealing that pod shell suffers from various adverse conditions when developing underground. Shell has evolved the capacity to adapt to underground conditions as a protective organ and perceive abiotic and biotic stress not only as damaging stimuli but also probably as signals for swelling. To further identify transcripts whose expression level increase, accompanying with pod enlarging, we require a more stringent criteria that the expression levels of transcripts are monotonically increasing from P2SH to P6SH while maintaining an average FPKM > 1 and two-fold changes. These transcripts were analyzed using KOBAS[15] to identify enriched metabolic pathways in which they function. Among them, four pathways were significantly enriched ($P < 0.01$) using a P-value based on hypergeometric distribution (**Table S10**). Enzymes for both pathways are important of regulating the partitioning of carbon into hemicellulose away from starch, sucrose and cellulose.

7. Additional specifications about Developmental dynamics of the seed transcriptome

To explore developmental dynamics of the seed transcriptome, we examined enrichment or depletion of up-regulated genes over the entire development by PageMan[16] functional category using a Fisher exact test ($P < 0.01$, $FDR < 0.05$).

Peanut seed development can be divided into two different periods. From our data sets corresponding to the two periods, we detected a total of 1,047 DE transcripts between them **(Table S24)**. To further explore the DE transcripts, we used MapMan annotation to assign genes to functional categories. Further analysis indicated that more than half of DE transcripts were preferentially activated in embryogenesis. Notably, the expression levels of approximately 25% of DE transcripts increased in P6SD to P9SD, but then dramatically decreased in P10SD, representing genes that were expressed at the low levels during desiccation.

8. Additional illustration of photosynthetic genes analysis in peanut pod

To confidently identify a set of transcripts whose transcription is activated before penetration into the soil, we imposed two criteria. First, we require that the transcripts in P0 should be DE transcripts in comparison to all subterranean developmental stages. Second, we require that the FPKM in P0 is at least twofold higher than the FPKM in each subterranean stage (P1 to P10). A total of 785 transcripts passed both filters (**Table S17**). Approximately 50.7% of these transcripts are also identified as aerial-pod-preferred transcripts in the previous study. The remaining 50% of transcripts might be due to material collection and sequencing coverage. All P0-preferred transcripts were analyzed using KOBAS 2.0[15] to identify the metabolic pathways in which they function in aerial pods. Consistent with the previous study, the pathway enrichment analysis of aerial pod-preferred transcripts revealed that photosynthetic genes are enriched in developing pod under light conditions (**Table** S**25**). To obtain detailed expression changes in individual developmental stages, Fisher's exact test was used to examine whether significantly more genes in photosynthesis functional categories at a given developmental stage were enriched in up-regulation when normalized to their average expression using PageMan[16].

References

[1] A. Voshall, E. N. Moriyama. Next-generation transcriptome assembly and analysis: Impact of ploidy. Methods. 2019.

[2] J. A. Martin, Z. Wang. Next-generation transcriptome assembly. Nat Rev Genet. 2011, 12(10): 671-682.

[3] G. Pertea, X. Huang, F. Liang, V. Antonescu, R. Sultana, S. Karamycheva, Y. Lee, J. White, F. Cheung, B. Parvizi, J. Tsai, J. Quackenbush. TIGR Gene Indices clustering tools (TGICL): a software system for fast clustering of large EST datasets. Bioinformatics. 2003, 19(5): 651-652.

[4] H. H. Chou, M. H. Holmes. DNA sequence quality trimming and vector removal[J]. Bioinformatics. 2001, 17(12): 1093-1104.

[5] S. Li, H. H. Chou. LUCY2: an interactive DNA sequence quality trimming and vector removal tool. Bioinformatics. 2004, 20(16): 2865-2866.

[6] W. Li, A. Godzik. Cd-hit: a fast program for clustering and comparing large sets of protein or nucleotide sequences. Bioinformatics. 2006, 22(13): 1658-1659.

[7] R. Garg, R. K. Patel, S. Jhanwar, P. Priya, A. Bhattacharjee, G. Yadav, S. Bhatia, D. Chattopadhyay, A. K. Tyagi, M. Jain. Gene discovery and tissue-specific transcriptome analysis in chickpea with massively parallel pyrosequencing and web resource development. Plant Physiol. 2011, 156(4): 1661-1678.

[8] B. J. Haas, A. Papanicolaou, M. Yassour, M. Grabherr, P. D. Blood, J. Bowden, M. B. Couger, D. Eccles, B. Li, M. Lieber, M. D. Macmanes, M. Ott, J. Orvis, N. Pochet, F. Strozzi, N. Weeks, R. Westerman, T. William, C. N. Dewey, R. Henschel, R. D. Leduc, N. Friedman, A. Regev. De novo transcript sequence reconstruction from RNA-seq using the Trinity platform for reference generation and analysis. Nat Protoc. 2013, 8(8): 1494-1512.

[9] M. H. Schulz, D. R. Zerbino, M. Vingron, E. Birney. Oases: robust de novo RNA-seq assembly across the dynamic range of expression levels. Bioinformatics. 2012, 28(8): 1086-1092.

[10] X. Chen, W. Zhu, S. Azam, H. Li, F. Zhu, H. Li, Y. Hong, H. Liu, E. Zhang, H. Wu, S. Yu, G. Zhou, S. Li, N. Zhong, S. Wen, X. Li, S. J. Knapp, P. Ozias-Akins, R. K. Varshney, X. Liang. Deep sequencing analysis of the transcriptomes of peanut aerial and subterranean young pods identifies candidate genes related to early embryo abortion. Plant Biotechnol J. 2013, 11(1): 115-127.

[11] P. Jiang, H. Wu, W. Wang, W. Ma, X. Sun, Z. Lu. MiPred: classification of real

and pseudo microRNA precursors using random forest prediction model with combined features. Nucleic Acids Res. 2007, 35(Web Server issue): W339-W344.

[12] Z. Ning, A. J. Cox, J. C. Mullikin. SSAHA: a fast search method for large DNA databases. Genome Res. 2001, 11(10): 1725-1729.

[13] C. Trapnell, B. A. Williams, G. Pertea, A. Mortazavi, G. Kwan, M. J. van Baren, S. L. Salzberg, B. J. Wold, L. Pachter. Transcript assembly and quantification by RNA-Seq reveals unannotated transcripts and isoform switching during cell differentiation. Nat Biotechnol. 2010, 28(5): 511-515.

[14] O. Thimm, O. Blasing, Y. Gibon, A. Nagel, S. Meyer, P. Kruger, J. Selbig, L. A. Muller, S. Y. Rhee, M. Stitt. MAPMAN: a user-driven tool to display genomics data sets onto diagrams of metabolic pathways and other biological processes. Plant J. 2004, 37(6): 914-939.

[15] C. Xie, X. Mao, J. Huang, Y. Ding, J. Wu, S. Dong, L. Kong, G. Gao, C. Y. Li, L. Wei. KOBAS 2.0: a web server for annotation and identification of enriched pathways and diseases. Nucleic Acids Res. 2011, 39(Web Server issue): W316-W322.

[16] B. Usadel, A. Nagel, D. Steinhauser, Y. Gibon, O. E. Blasing, H. Redestig, N. Sreenivasulu, L. Krall, M. A. Hannah, F. Poree, A. R. Fernie, M. Stitt. PageMan: an interactive ontology tool to generate, display, and annotate overview graphs for profiling experiments. BMC Bioinformatics. 2006, 7: 535.
