# Supplementary material for: Global transcriptome analysis of subterranean pod and seed in peanut (Arachis hypogaea L.) unravels the complexity of fruit development under dark condition
Source: Sci Rep. 2020 Aug 3;10:13050. doi: 10.1038/s41598-020-69943-7 (PMC7398922; doi:10.1038/s41598-020-69943-7)
Supplement: Supplementary file 2 — Supplementary figures. [file 41598_2020_69943_MOESM2_ESM.pdf]

Global transcriptome analysis of subterranean pod and seed in peanut (*Arachis hypogaea* L.) unravels the complexity of fruit development under dark condition

Hao Liu[1], Xuanqiang Liang[1], Qing Lu[1], Haifen Li[1], Haiyan Liu[1], Shaoxiong Li[1], Rajeev Varshney[2], Yanbin Hong[1*], Xiaoping Chen[1*]

[1]Guangdong Provincial Key Laboratory of Crop Genetic Improvement, Crops Research Institute, Guangdong Academy of Agricultural Sciences (GAAS), Guangzhou 510640, China.

[2]International Crops Research Institute for the Semi-Arid Tropics (ICRISAT), Patancheru 502324, India.

* Corresponding. chenxiaoping@gdaas.cn, hongyanbin@gdaas.cn.

Appendix B. supplementary figures

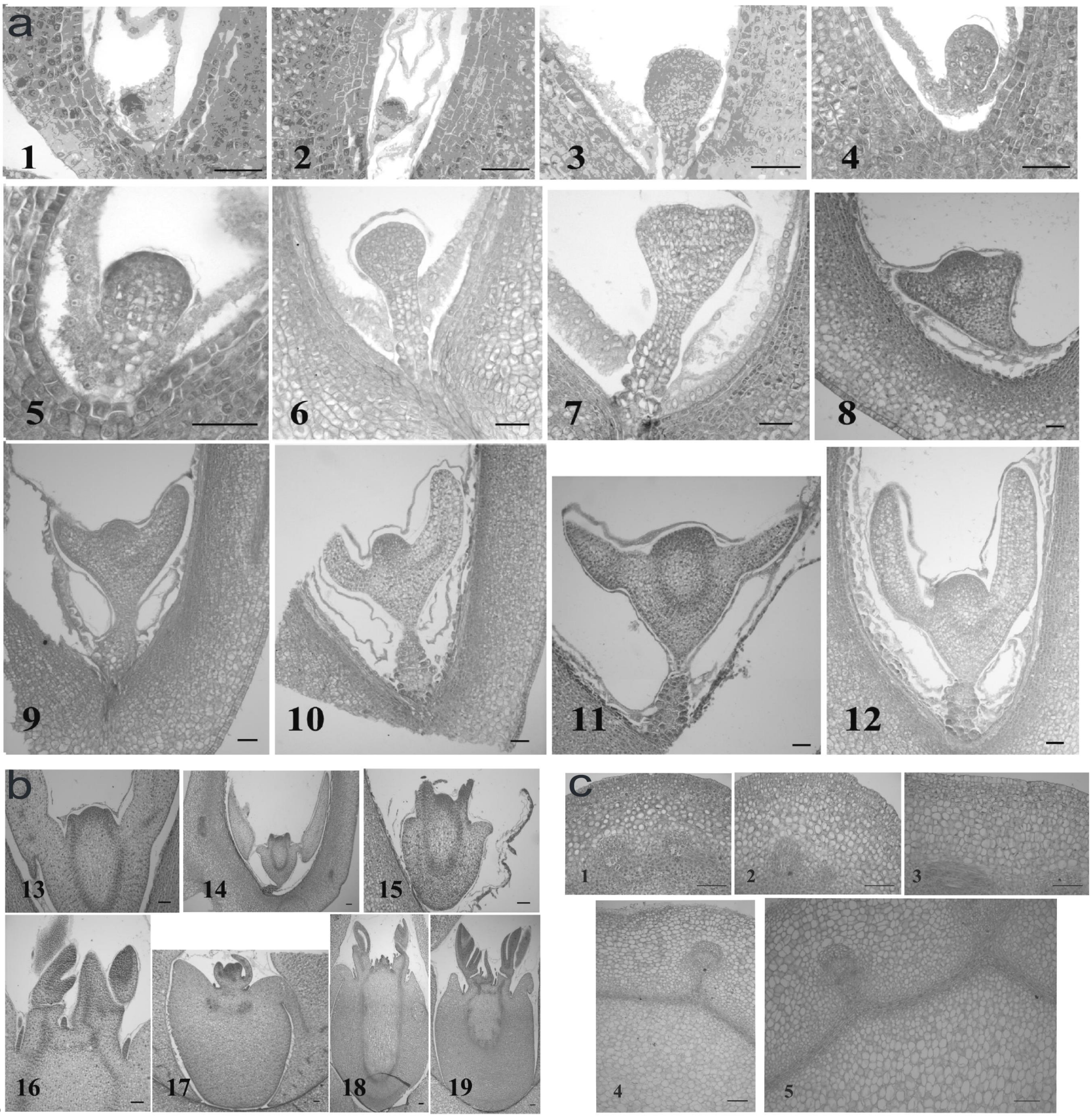

Figure. S1 Light micrographs of the developing embyos along peanut pod development. (**a**) Developing embryos (1-12) during pod expansion with 1-12 mm of pod size and 1-2 mm of seed size. Globular (1-6), heart (7, 8) embryo, embryos with 1st and 2nd cotyledons appear (8, 9), embryos with cotyledons elongation (10-12). (**b**) Developing embryos (13-19) during seed filling with 2.5 to 8 mm of seed size. Appearance of the first pair of leaf primordia (13-15), leaf primordial elongation (15, 16), development of the second pair of leaf primordial (17-19). According to embryo development, we separate/split pod devilment into 11 stages, P0 (1 or 2), P1 (1 or 2), P2 (2-4), P3 (4-7), P4 (7-10), P5 (10-12), P6 (13-15), P7 (15-17), P8 (17-19), P10 and P11 (mature and desiccation). Bars is 50 μm. (**c**) Microsection observation of developing pod along with its size (diameter) grown from 1mm to 5 mm, the number 1-5 in separate panels represented the pod size from 1 mm to 5 mm, respectively. The number and size of cells also increased. Bars is 100 μm.

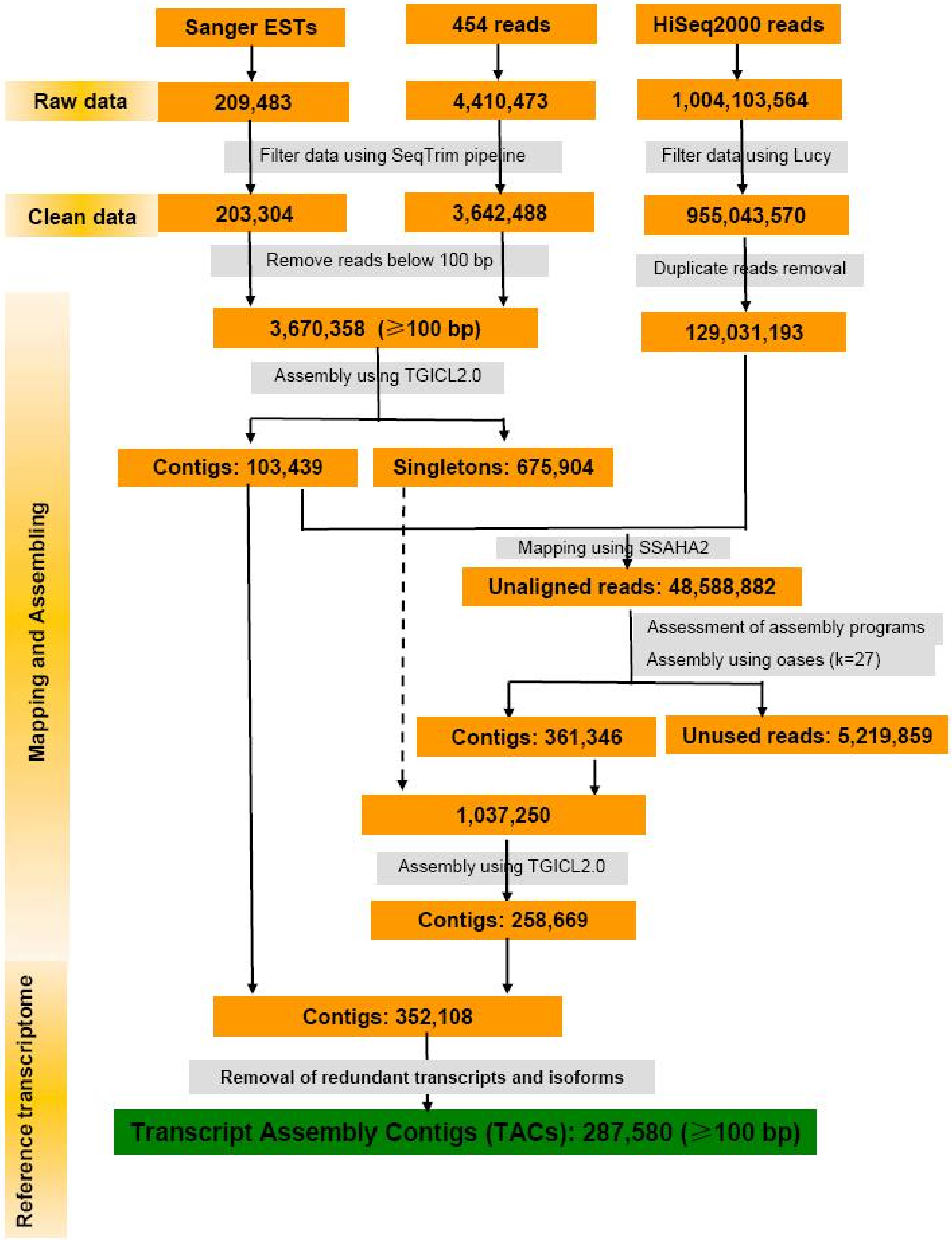

Figure. S2 Overall strategy for the combined assembly of the peanut reference transcriptome

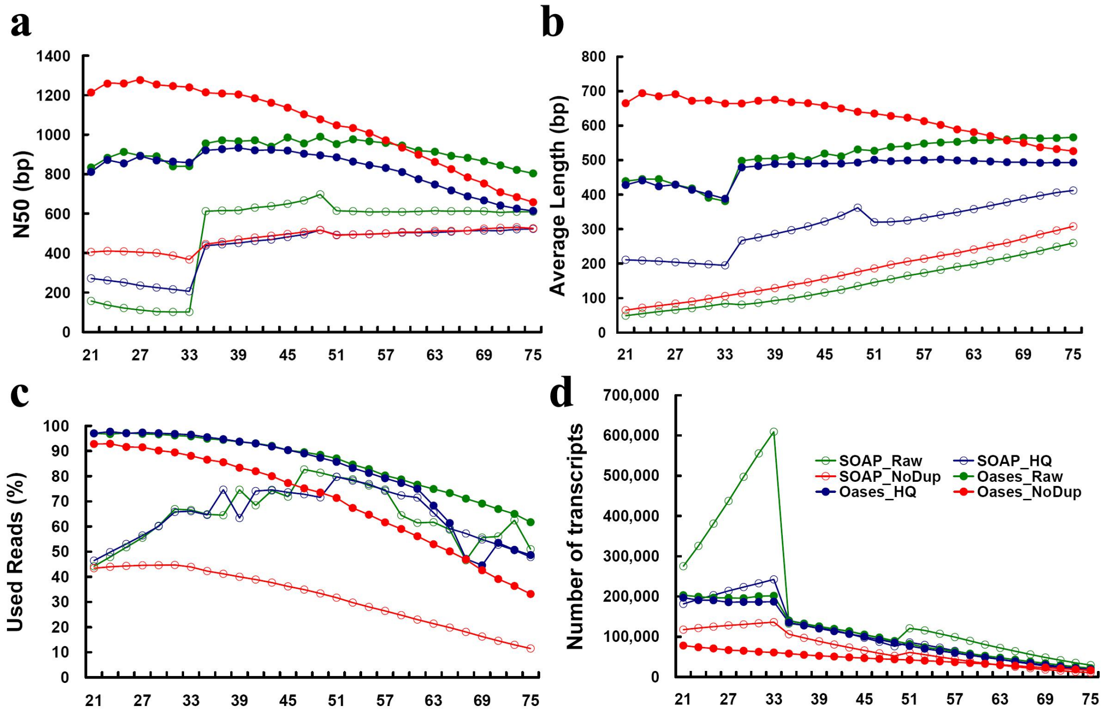

Figure. S3 Comparison of assembly of P0 data set using SOAP and Oases programs as a function of k-mer length. The N50 length (a), average length (b), number of used reads (c) and number of transcripts (d) were compared for assemblies using two programs. The data sets include raw data (Raw, indicated in green), high-quality data (HQ, in blue) and high-quality data with duplicate reads removed (NoDup, in red). The solid circles and hollow circles indicate assemblies using SOAP and Oases, respectively.

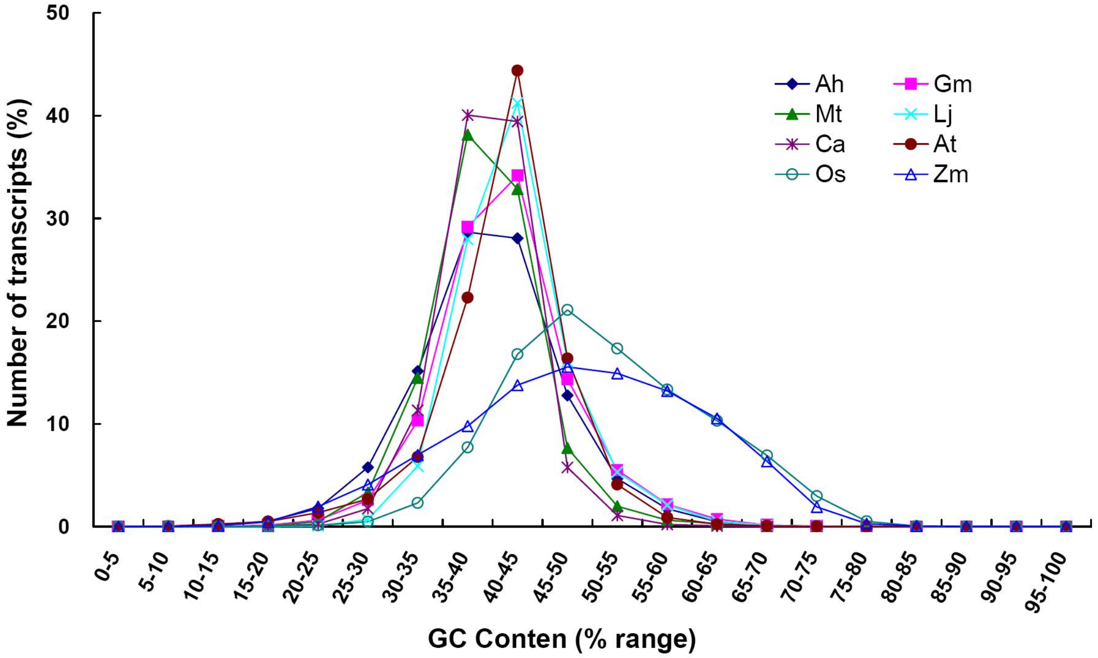

Figure. S4 GC content analysis of peanut transcripts. The percentage of transcripts with GC content within a 5% interval range are represented for peanut (Ah), soybean (Gm), *Medicago truncatular* (Mt), *Lotus japonicus* (Lj), chickpea (Ca), *Arabidopsis* (At), rice (Os) and maize (Zm).

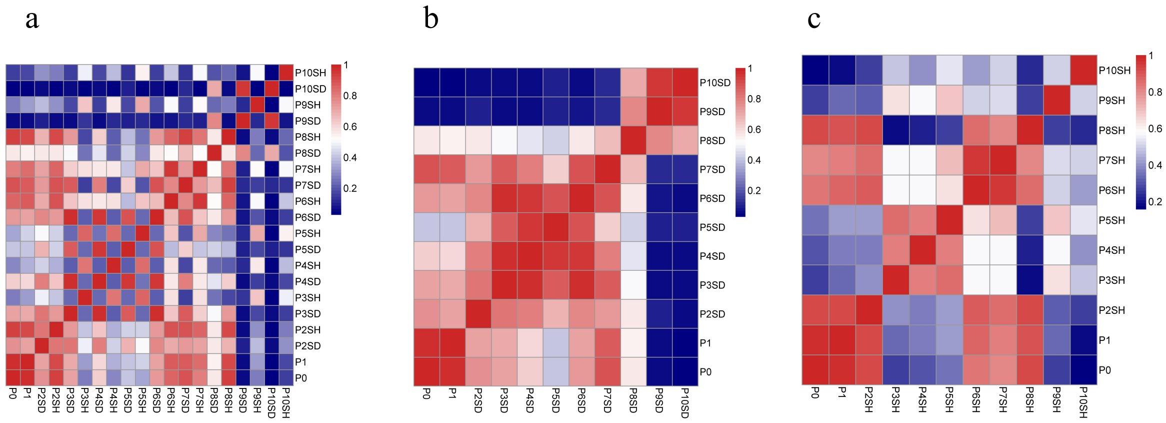

Figure. S5 Correlation of gene expression levels between samples using RNA-seq data. Each developmental stage is highly correlated with its adjacent stage. A low correlation is found between seed and shell tissues. Red, high correlation; blue, low. (a) All samples. (b) P0, P1 and seed (SD) samples. (c) P0, P1 and shell (SH) samples

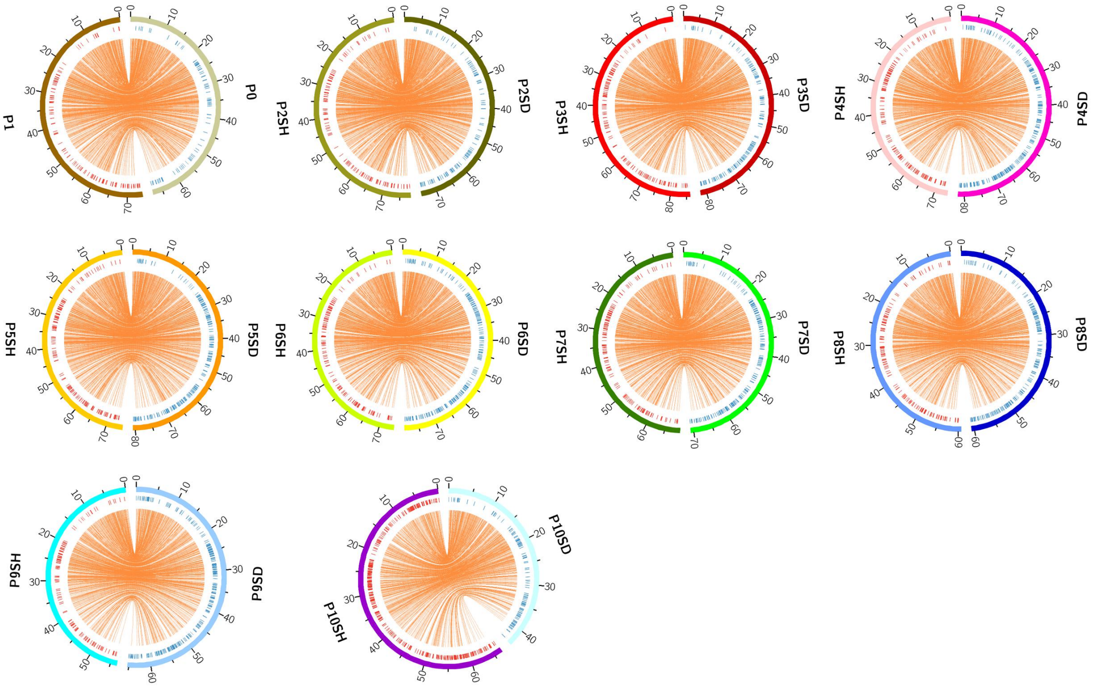

Figure. S6 Tissue- and stage-specific as well as shared TFs across peanut pod development. Orange links indicated shared TFs between seed and shell tissues at the same stages. Highlighted bands (red and blue) show specific TFs in seed ir shell.

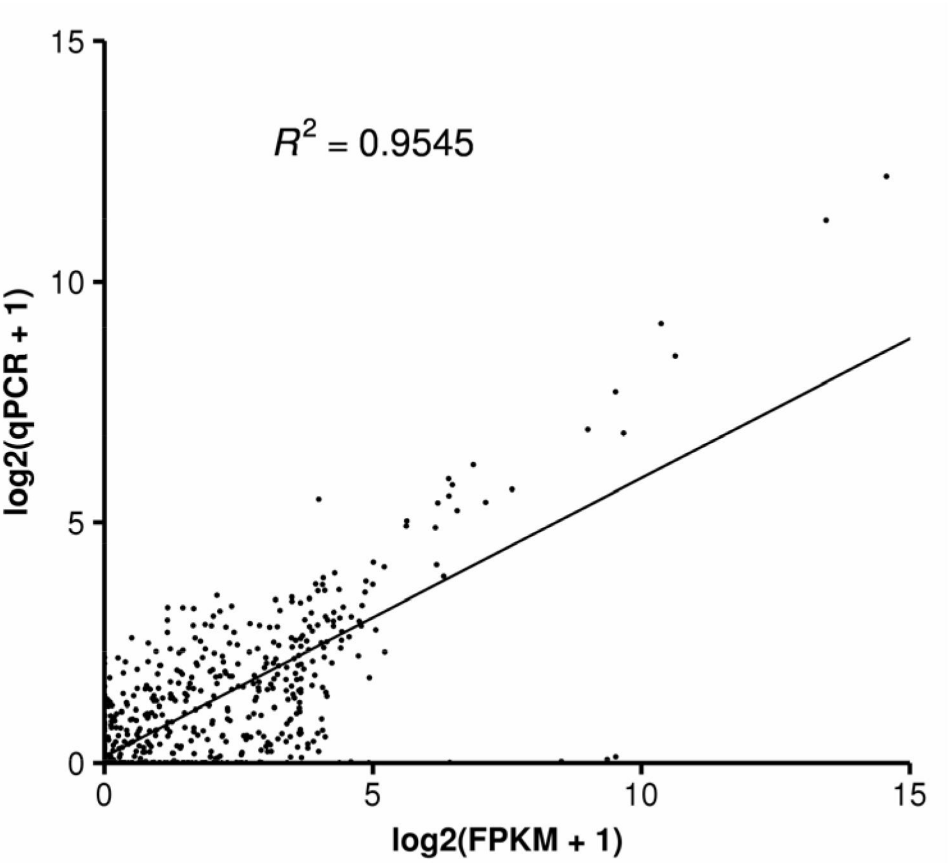

Figure. S7 Validation of the RNA-seq results by qRT-PCR. A comparison of the expression levels measured by RNAseq and qRT-PCR was conducted for 30 randomly selected transcripts in 20 samples.

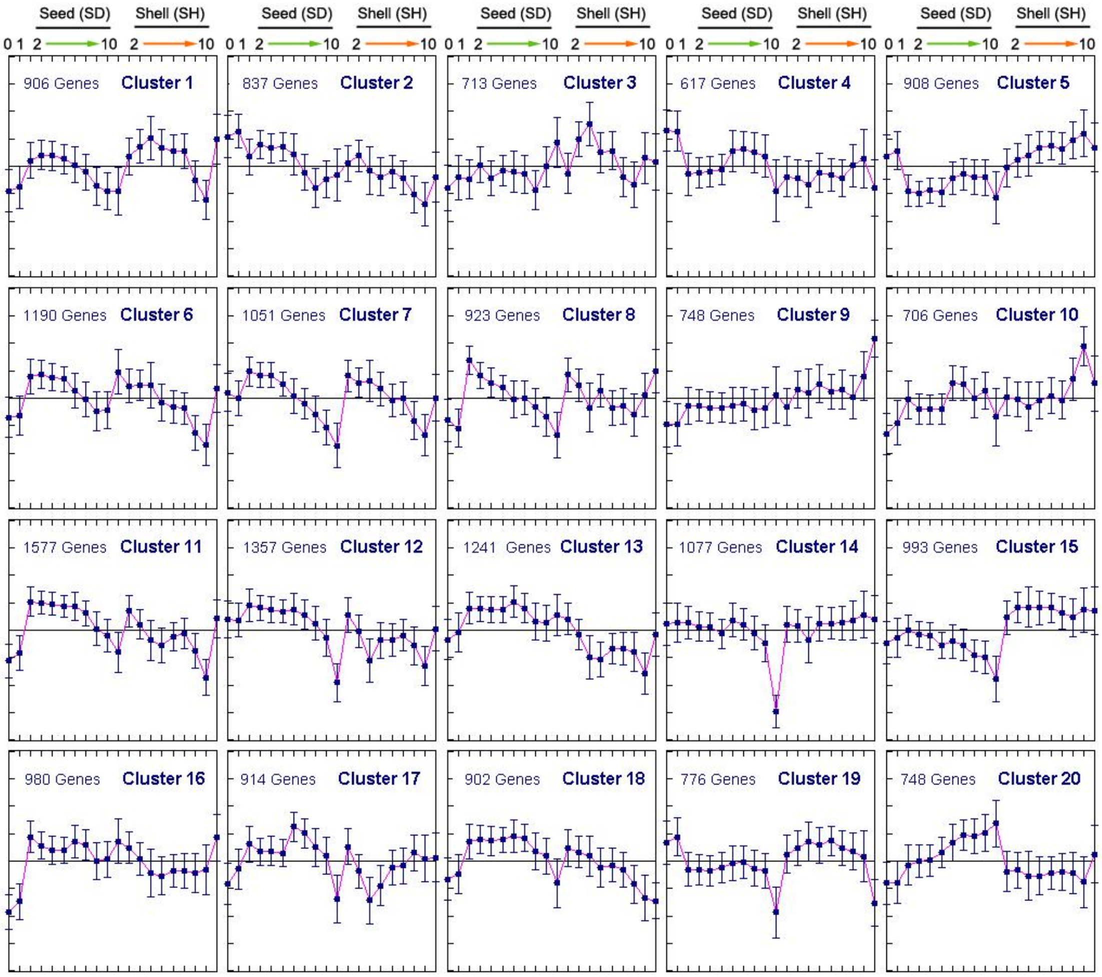

Figure. S8 K-means clusters of co-expressed transcripts. Twenty clusters were identified along pod development (P0, P1, P2SD-P10SD, and P2SH-P10SH). Error bars show standard deviation.

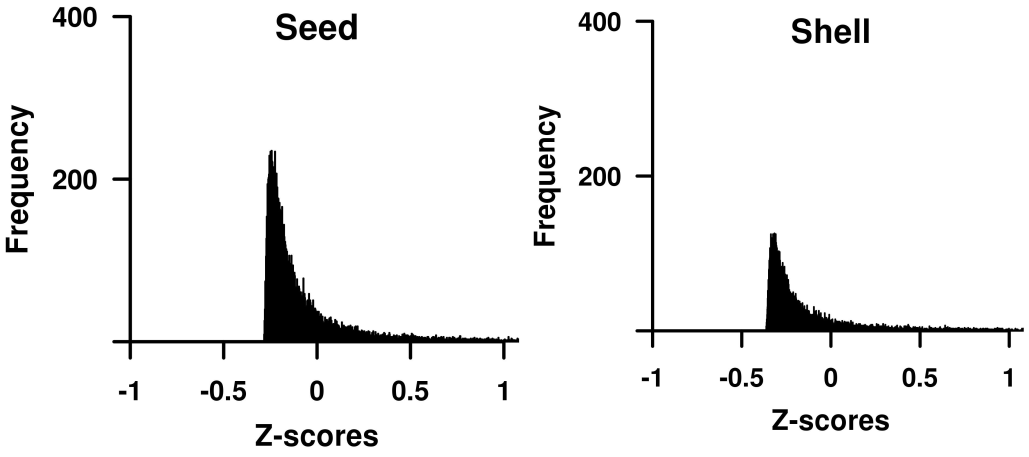

Figure. S9 Histograms of relative expression levels (Z scores) of co-expressed transcripts in seed and shell tissues. Expression levels in seed and shell show a shift towards to low values relative to their overall average levels.

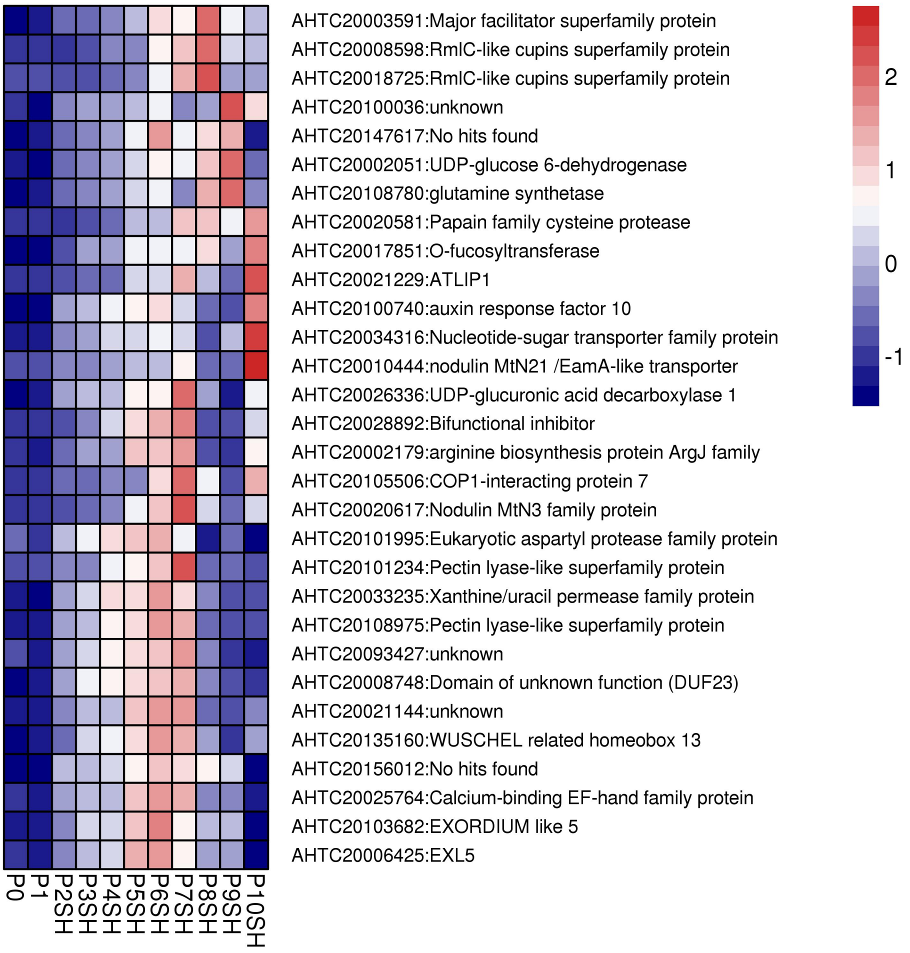

Figure. S10 Expression profiles of differentially expressed genes whose expression levels are monotonically increasing pod expansion (from P2SH to P6SH).

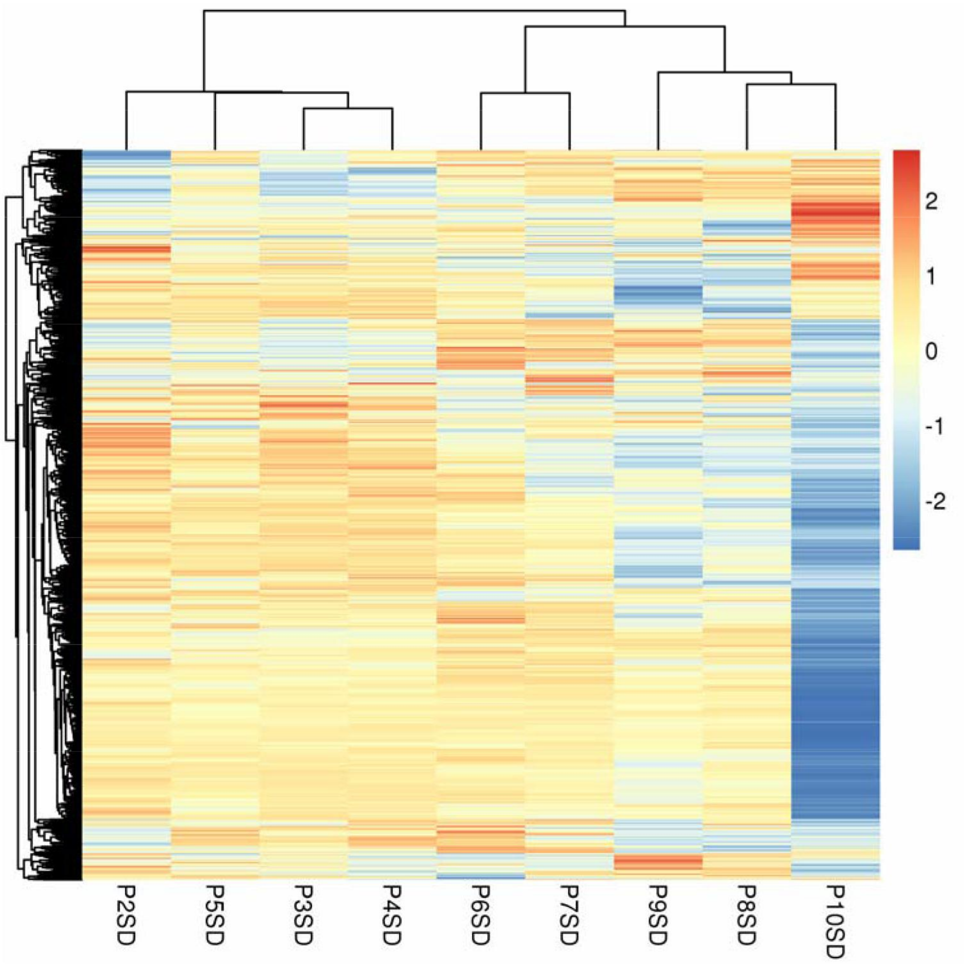

Figure. S11 Hierarchical clustering analysis of 1475 differentially expressed transcription factors from P2SD to P10SD showed that seed development stages (from P2 to P10) fall into two major clusters representing embryogenesis (P2SD to P5SD) and seed filling and desiccation (P6SD to P10SD).

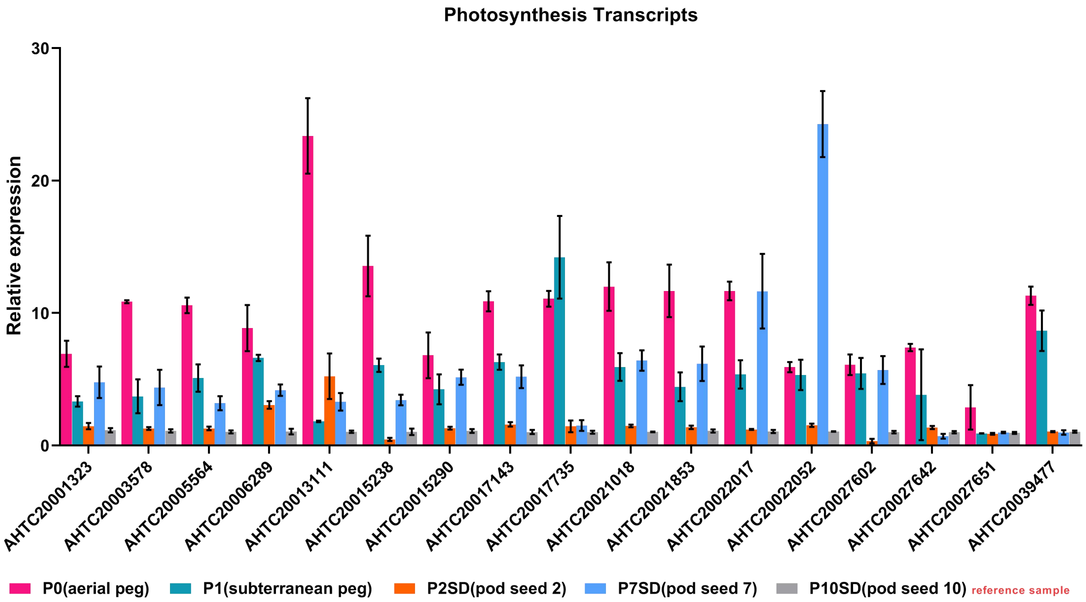

Figure S12. qRT-PCR validated the relative expression levels of seventeen transcripts related to photosynthesis at five time-points in developing seed. P10SD as the reference sample, values shown are means ± SD of three independent repeats.

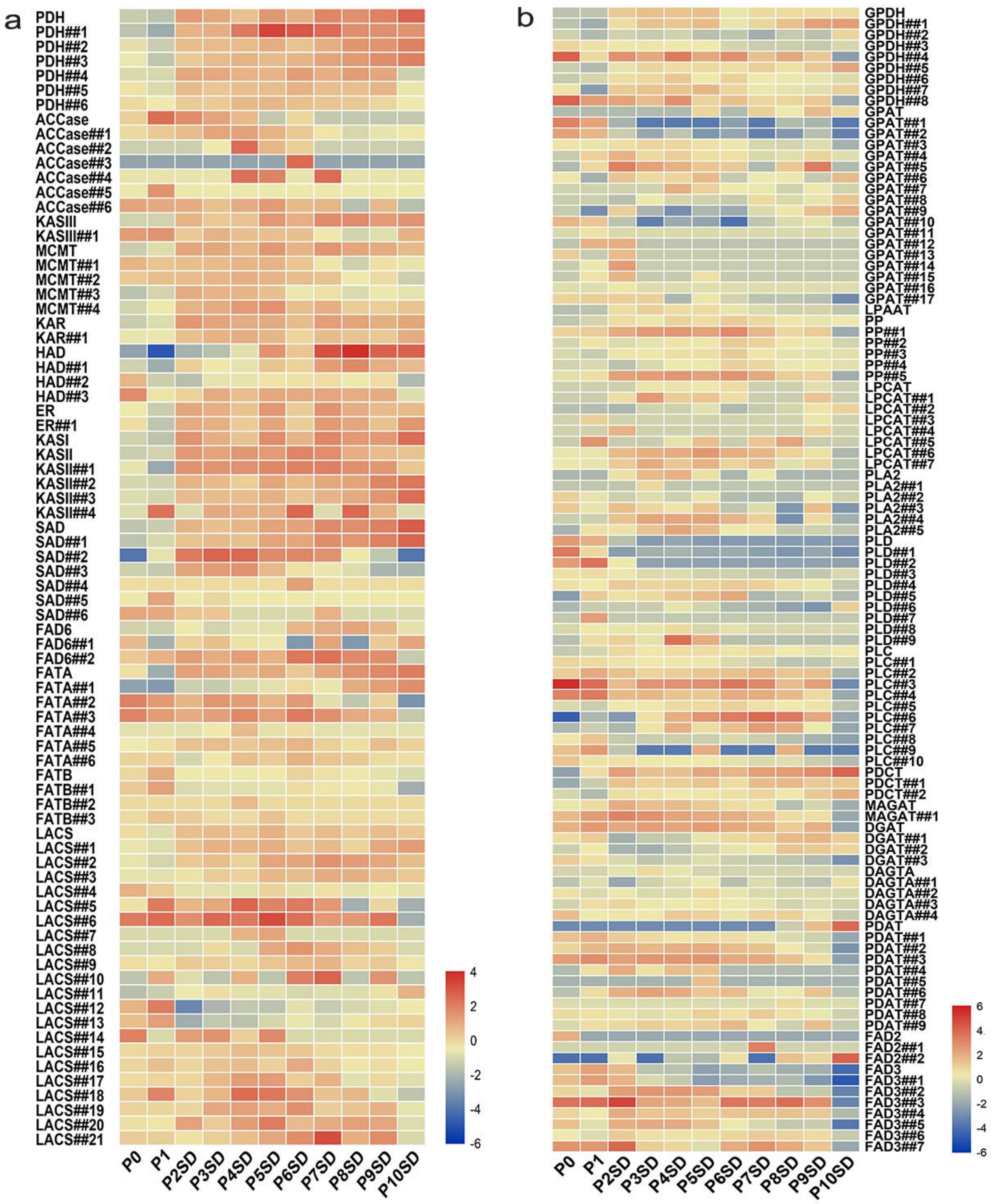

Figure S13. Heatmap displayed the fatty acid synthesis genes and TAG metabolism genes during the seed development (**Table S26**). (**a**) 78 transcripts regulated the fatty acid de novo synthesis and elongation. (**b**) 104 transcripts involved into the regulation of TAG metabolism pathway.
